# A Simple Method for Analyzing Exome Sequencing Data Shows Distinct Levels of Nonsynonymous Variation for Human Immune and Nervous System Genes

**DOI:** 10.1371/journal.pone.0038087

**Published:** 2012-06-06

**Authors:** Jan Freudenberg, Peter K. Gregersen, Yun Freudenberg-Hua

**Affiliations:** 1 Robert S. Boas Center for Human Genetics and Genomics, The Feinstein Institute for Medical Research, Northshore LIJ Healthsystem, Manhasset, New York, United States of America; 2 Department of Psychiatry, Division of Geriatric Psychiatry, Northshore LIJ Healthsystem, Glen Oaks, New York, United States of America; 3 Litwin-Zucker Center for Alzheimer’s Disease and Memory Disorders, The Feinstein Institute for Medical Research, Northshore LIJ Healthsystem, Manhasset, New York, United States of America; Institut Pasteur, France

## Abstract

To measure the strength of natural selection that acts upon single nucleotide variants (SNVs) in a set of human genes, we calculate the ratio between nonsynonymous SNVs (nsSNVs) per nonsynonymous site and synonymous SNVs (sSNVs) per synonymous site. We transform this ratio with a respective factor *f* that corrects for the bias of synonymous sites towards transitions in the genetic code and different mutation rates for transitions and transversions. This method approximates the relative density of nsSNVs (*rdnsv*) in comparison with the neutral expectation as inferred from the density of sSNVs. Using SNVs from a diploid genome and 200 exomes, we apply our method to immune system genes (ISGs), nervous system genes (NSGs), randomly sampled genes (RSGs), and gene ontology annotated genes. The estimate of *rdnsv* in an individual exome is around 20% for NSGs and 30–40% for ISGs and RSGs. This smaller *rdnsv* of NSGs indicates overall stronger purifying selection. To quantify the relative shift of nsSNVs towards rare variants, we next fit a linear regression model to the estimates of *rdnsv* over different SNV allele frequency bins. The obtained regression models show a negative slope for NSGs, ISGs and RSGs, supporting an influence of purifying selection on the frequency spectrum of segregating nsSNVs. The y-intercept of the model predicts *rdnsv* for an allele frequency close to 0. This parameter can be interpreted as the proportion of nonsynonymous sites where mutations are tolerated to segregate with an allele frequency notably greater than 0 in the population, given the performed normalization of the observed nsSNV to sSNV ratio. A smaller y-intercept is displayed by NSGs, indicating more nonsynonymous sites under strong negative selection. This predicts more monogenically inherited or de-novo mutation diseases that affect the nervous system.

## Introduction

A thorough understanding of sequence variation of human genes is important to understand the molecular basis of human disease. Many earlier studies had analyzed single nucleotide variants (SNVs) in coding regions of human genes and demonstrated a signature of purifying selection on nonsynonymous SNVs (nsSNVs) by comparing their frequencies to synonymous SNVs (sSNVs) [1]–[9]. Since then, the size of SNV datasets has increased tremendously [10], [11]. In the present manuscript, we quantify the level of nsSNVs in different sets of genes as normalized by the levels of sSNVs, using coding SNVs from two published whole exome datasets [12], [13]. We specifically focus on genes with molecular roles in the immune and nervous system. On the phenotype level, functional variants in these genes are most likely to impact immune and nervous system traits. Insights into the patterns of genetic variation in these genes therefore sheds light on the genetic architecture of immune and nervous system phenotypes.

Both the immune and the nervous system consist of a large number of specialized cell types that function in strong interaction with the environment. Comparative genomic studies show that both human immune and nervous system genes are outliers in terms of their sequence evolution. While immune system genes are generally fast evolving genes [14], [15] in mammals, nervous system genes are highly conserved, but show accelerated evolution in hominids [15], [16]. The phylogenetic distribution of the respective genes shows major genetic innovations in early vertebrates. Nervous system gene families typically exist in invertebrates [17], but overproportionally expanded in early vertebrates [18]–[20]. In contrast, gene families underlying innate immunity arose before the vertebrate-invertebrate split [21], whereas families underlying adaptive immunity arose after this split [22]. With respect to more recent human evolution, immune and nervous system genes are known to display the least linkage disequilibrium and the highest recombination rates in the human genome [23]–[25].

Taken together, these earlier results indicate that both human immune and nervous system genes are particularly exposed to various evolutionary forces. Accordingly, it is interesting to specifically ask how immune and nervous system genes compare to each other and to other genes with respect to their levels of functional variation in the human genome. To address this question we obtain two sets of nervous system genes (NSGs) and immune system genes (ISGs) based on expression data [26] and keyword database search [27]. We further compare these broadly defined sets of NSGs and ISGs with randomly sampled genes (RSGs) and with gene ontology (GO) annotated genes. To quantify the level of single nucleotide variation in a set of genes, we define the relative density of nonsynonymous variants (*rdnsv*) as compared with the neutral expectation that is inferred from the density of synonymous variants. We find that *rdnsv* varies among gene sets as well as among individual exomes and over SNV allele frequencies. Based on the on the change of *rdnsv* over the allele frequency of underlying SNVs, we predict greater proportions of strongly deleterious sites for NSGs, which can explain more monogenic phenotype manifestations for the nervous system as well as a greater importance of de-novo mutations. Our analysis further supports widespread purifying selection on segregating nsSNVs for nearly all groups of genes, including both NSGs and ISGs. Thus, mildly deleterious nsSNVs are likely to play a causative role in both groups of disease phenotypes. However, certain sets of ISGs display a greater *rdnsv* estimate than the genomic background, which supports the idea of an enhanced influence of positive selection on their frequency spectrum of functional nsSNVs and putative disease variants. This observation can explain a possible presence of more common disease variants for the immune system.

## Methods

### Analysis of Coding Sequence Variation

If natural selection would equally act on nonsynonymous as on synonymous mutations, the value of *rdnsv* for a set of genes were expected to equal 1. Vice versa, *rdnsv* were expected to equal 0, if selection would not tolerate any nonsynonymous mutations to segregate in the population at all. In reality, for most sets of gene the value of *rdnsv* is likely to be between 0 and 1 and expected to be shaped by natural selection in two ways: 1) by the proportion of nsSites where any nsSNV with a frequency notably greater than 0 are not tolerated to segregate, and 2) by the effect of weak selection on segregating nsSNVs at any of the remaining nsSites. In the following we describe our approach to disentangle these two factors based on an approximate estimate of *rdnsv* for a set of human genes and chromosomes.

In order to derive a simple estimate of *rdnsv*, we view synonymous sites (sSites) and nonsynonymous sites (nsSites) as mutational opportunities. We calculate the number sSites and nsSites in a set of genes as the sum over all possible single basepair changes in the reference sequence based on the standard genetic code. The total number of sites in a coding sequence is the sum over all its sSites and nsSites. We use the coding regions from the human genome assembly as reference sequence for each gene. Nucleotides at partially degenerate sites with two synonymous and one nonsynonymous mutational opportunities are counted as 2/3 sSite and 1/3 nsSite, whereas nucleotides with one synonymous and two nonsynonymous mutational opportunities are counted as 1/3 sSite and 2/3 nsSite. Nucleotides at fourfold degenerate positions are counted as 3/3 sSite, whereas nucleotides at non-degenerate positions are counted as 3/3 nsSite. We normalize the observed numbers of nsSNVs and sSNVs by calculating the rate of nsSNVs per nsSite (*Rn*) and the rate of sSNVs per sSite (*Rs*), e.g. *Rn = nsSNV/nsSite* and *Rs = sSNV/sSite*. However, the genetic code is enriched for transitions among synonymous changes and transition mutations are more likely to occur than transversion mutations. To estimate the relative density of nsSNVs as compared with the neutral expectation (*rdnsv)* as inferred from the density of sSNVs, we therefore need to correct the *Rn*/*Rs* ratio for the influence of the genetic code on synonymous and nonsynonymous mutation rates.

Here we approach this task by deriving a respective correction factor *f* from the observed numbers of transition and transversion sites that are synonymous or nonsynonymous sites as compared with the respective numbers of sites that were expected for a random genetic code. Based on our definition of sites as mutational opportunities, we can view each basepair as 1/3 transition site and 2/3 transversion site, because it allows for one transition and two transversion mutations. The number of transition sSites (*sSite_ts_*) can then be calculated by summing up all opportunities for single basepair mutations in the reference sequence that are both synonymous and transitions. Analogously, we can calculate the numbers of transition nsSites (*nsSite_ts_*), transversion sSites (*sSite_tv_*) and transversion nsSites (*nsSite_tv_*). One may now assume that at any specific basepair a transition mutation is 4 times more likely to occur than a transversion mutation during an arbitrary time unit, based on a 2-fold higher rate per basepair for transition mutations (i.e. a transition/transversion mutation rate ratio of 2) and each basepair allowing for only 1 transition, but 2 transversion mutational opportunities. We can then adjust the *Rs* values based on the ratio between observed and expected proportions of transitions among sSites. More formally we can write:
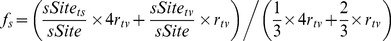
(1)where *r_tv_* denotes the rate of transversion mutations (with 4 *r_tv_* = *r_ts_*), and *sSite_ts_*/*sSite* and *sSite_tv_*/*sSite* denote the proportions of sSites that are transition or transversion sites.

In the reference genome sequence, we observe (in all evaluated gene categories) that transition sites constitute close to 1/2 of all sSites (i.e. *sSite_ts_/sSite* ∼ 0.5 instead of a ratio of *sSite_ts_/sSite* = 1/3 that were expected for randomly distributed transition and transversion sites) (Table S1). Based on these observed counts of sites, this leads to *f_s_* = 1.25, which indicates a 25% increase of the synonymous mutation rate that is attributable to the structure of the genetic code. Thus, the rate of sSNVs per sSite that would be expected with a random genetic code can be obtained by dividing the observed *Rs* value by *f_s_*. The fraction of sSites that are transition and transversion sites is very similar across our sets of candidate genes (Table S1).

To compensate for the increased proportion of transition sites among sSites a corresponding decreased proportion must exist among nsSites. Accordingly, we further observe in the human genome reference sequence a proportion of 4/14 of nsSites being transition sites (i.e. *nsSite_ts_*/*nsSite* ∼0.285) instead of the expected 1/3. (Table S1). When applying now the analog reasoning for nsSites as above for sSites, this leads to an estimated overall reduction of the nonsynonymous mutation rate by 7.5% due to the structure of the genetic code. Thus, the observed *Rn* values need to be divided by the factor *f_ns_* = 0.925 to estimate the rate of nsSNVs per nsSite that would be expected with a random genetic code. In combination with the above correction factor *f_s_*, one may therefore estimate that the structure of the genetic code reduces the nonsynonymous mutation rate relative to the synonymous mutation rate by the factor:
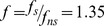
(2)Thus, multiplication of an observed *Rn*/*Rs* ratio by *f* allows for a correction for the biased structure of the genetic code.

Accordingly, we can now estimate the relative density of nonsynonymous variants:
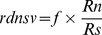
(3)Based on the above definition of *Rn* and *Rs* this is furthermore equivalent to:



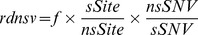
(4)Because the ratio *sSite*/*nsSite* is ∼0.3 for the gene sets analyzed here (Table S1), a simple rule of thumb to approximate *rdnsv* for large gene sets is given by multiplying the ratio of *nsSNV*/*sSNV* by f ’ = 0.4 (≅ 0.3 *f* ).
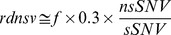
(5)


To analyze the dependency of *rdnsv* on the allele frequency of SNVs, we calculate *rdnsv* separately for different allele frequency bins of SNVs (frequency below 2%, 5%, 10%, 20%, 40%, 80% and 95%). We then fit a linear regression model to the values of *rdnsv* over the allele frequency. We logarithmically transform the allele frequency variable, because the frequency spectrum of human SNVs is known to be strongly shifted towards rare variants [12], [28]. In the fitted regression model, two predicted values of *rdnsv* deserve particular attention: First, the y-intercept (*rdnsv_0_*), which gives the relative density of nsSNVs for an allele frequency near 0. The estimate of *rdnsv_0_* thus approximates the proportion of nonsynonymous mutations that are not immediately rejected by selection out of all nonsynonymous mutations, assuming that sSNVs evolve neutrally and the normalization of the ratio *nsSNV*/*sSNV* for different underlying nonsynonymous and synonymous mutation rates. This proportion equals the proportion of nonsynonymous mutations that occur on neutral nsSites or weakly deleterious nsSites, which is the proportion of nsSites where nsSNVs are tolerated to segregate in the population. Vice versa, 1- *rdnsv_0_* (the difference between the expected intercept under the absence of selection and the observed intercept) approximates the proportion of nsSites (out of all nsSites), where nsSNVs are not tolerated to segregate even at very low frequency. The second predicted value of interest (*rdnsv_1_*) is the relative density of nsSNVs near the allele frequency 1. This value *rdnsv_1_* approximates the proportion of nsSites where purifying selection does not prevent nsSNVs from reaching fixation. Thus, estimating *rdnsv_0_* and *rdnsv_1_* for a set of genes may help to separate the influence of selection at strongly deleterious versus mildly deleterious and neutral sites. Both factors contribute to the overall value of *rdnsv* in a sample of chromosomes, but only the latter influences the allele frequency spectrum of nsSNVs.

We first apply our method to SNVs from a diploid European genome sequence as produced by traditional Sanger sequencing, which are obtained from the annotation track pgVenter in the UCSC genome database [13], [29]. We then apply the method to whole exome SNV data from 200 Danish individuals that are downloaded from the Beijing Genomics Institute (BGI) website [12]. We calculate the derived allele frequency based on the status of chimp alleles, which we obtain from the human-chimp BlastZ alignment in the UCSC database. If no chimp allele was available, the minor allele was taken as the most likely derived allele (which applies to 2.6% of the SNVs). Those SNVs from the exome dataset that cannot be lifted to the NCBI37 assembly with the UCSC liftOver tool are excluded. In both SNV datasets, sSNVs and nsSNVs are defined based on the positions of SNVs in the UCSC gene models. Both missense and nonsense SNVs are counted as nsSNVs.

### Definition of Candidate Genes

Transcripts for human autosomal genes are obtained from the ‘knownGenes’ and ‘knownCanonical’ annotation tracks of the University of California Santa Cruz (UCSC) genome database [29]. Genes with specific roles in the immune system (in the following referred to as ISGs) and the nervous system (in the following referred to as NSGs) are obtained based on the GNF2 expression dataset [26]. The 1500 genes specifically expressed in the greatest number of immune tissues and the 1500 genes specifically expressed in the greatest number of nervous system tissues, respectively, are taken as candidate genes. Because these sets of candidate genes are defined by the specific expression in disjunctive sets of tissues, no great overlap would be expected. Consistently, only four genes (*EVI2A*, *DOCK10*, *C9orf103*, *PTK2B*) belong to both sets and are removed from the subsequent analysis. Expression analyses for 16185 autosomal human genes are based on 62 out of the 79 tissues from the GNF2 dataset represented in UCSC database (table gnfHumanAtlas2Median), excluding disease, compound and fetal tissues [26]. Raw expression values are log2 transformed and for each gene subsequently normalized by a Z-score transformation across all included tissues. Genes with Z-scores greater 1.0 are considered as specifically expressed in a tissue. Nervous system tissues are defined by the labels temporal lobe, parietal lobe, occipital lobe, prefrontal cortex, cingulated cortex, cerebellum, cerebellum peduncles, amygdala, hypothalamus, thalamus, subthalamic nucleus, caudate nucleus, globus pallidus, olfactory bulb, pons, medulla oblongata, spinal cord, ciliary ganglion, trigeminal ganglion, superior cervical ganglion, dorsal root ganglion. Immune tissues are defined by the labels thymus, tonsil, lymph node, BM CD33pos myeloid, PB BDCA4pos dentritic cells, PB CD14pos monocytes, PB CD56pos NKCells, PB CD4pos Tcells, PB CD8pos Tcells, PB CD19pos Bcells. We find nervous system candidates specifically expressed in an average of 10.5 out the 21 nervous system tissues, 0.27 immune system tissues and 1.7 out of the remaining 31 other tissues. Immune system candidates are specifically expressed in an average of 7 out of the ten immune tissues, 0.6 nervous system tissues and 3.6 other tissues. Thus, both groups of candidate genes show roughly the same level of overall expression specificity, making a confounding effect of this variable unlikely. We additionally compare these functional candidate genes to a disjoint and equally sized set of random ‘non-candidate genes’, which all have GNF2 expression annotations.

A second set of functional candidate genes is obtained by keyword search with the term ‘(neuronal* or glial* or neural* or neurite or axon) and not olfactory’ and the term ‘immune* or immunological*’from Entrez Gene [27] (version 09/10). The retrieved genes are linked to known UCSC genes by their official gene names. This keyword search of gene annotations provides us with a list of 1334 ISGs and a list of 1817 NSGs. Of these genes, 294 genes are contained in both lists of keyword-based candidates and therefore removed from the analysis. A total of 367 genes from the remaining 1523 keyword-based NSGs is also included in the above list of expression-based NSGs, whereas a total of 181 from the remaining 1040 keyword-based ISGs is also included in the above expression-based ISGs.

The statistical comparison of gene categories is based on 10.000 permutations of the category status and counting how often a greater difference of the test statistic (e.g. *Rn*, *Rs* or *Rn/Rs*) is observed in the permuted than the observed data. The expected background values are calculated as the 2.5% and 97.5% quantiles from 10.000 sets of 1500 randomly sampled genes.

Monogenic disease gene annotation are obtained from the ‘morbidmap’ file of the Online Mendelian Inheritance in Men (OMIM) database (version 03/10). Complex disease locus annotations come from the file ‘GWASCatalog’ from the National Human Genome Research Institute (NHGRI) Genome Wide Association Study (GWAS) catalog website (version 03/10). The morbidmap and GWASCatalog files are manually searched for entries linked to immune or nervous system phenotypes. All complex disease loci from OMIM are discarded. Also GWASCatalog loci with P>5*10^−8^ are discarded. Nervous system phenotypes are defined as those affecting the nervous system, including both psychiatric and neurological disorders. Immune phenotypes are defined as those that relate to dysfunction of the immune system, including autoimmune disease and susceptibility to infection. Gene ontology (GO) annotations of human genes are obtained from the GO- database. [30].

## Results

### Relative Density of nsSNVs in Individual Exomes

We start by looking at SNVs in nervous system genes (NSGs) and immune system genes (ISGs) from the exome of an individual diploid genome sequence [13], with NSGs and ISGs being defined by their specific expression in the respective tissues. These expression-based candidates are further compared with an equally sized set of randomly sampled genes (in the following referred to as RSGs), which do not overlap with ISGs and NSGs. To evaluate the level of SNVs in these candidate genes, their different coding sequence length needs to be considered (Figure S1a. Therefore, we calculate the rate of nsSNVs per nsSite (*Rn*) and the rate of sSNVs per sSite (*Rs*) for each set of genes (Table S2a). We further normalize *Rn* by *Rs* and find that NSGs have a significantly reduced *Rn*/*Rs* ratio as compared with ISGs and RSGs (P<10^−5^). The *Rn*/*Rs* ratio accounts for possible differences read coverage, SNV call rates or mutation rate between gene categories, because these factors would equally affect the density of nsSNVs and sSNVs as measured by *Rn* and *Rs* in a gene category.

**Figure 1:**
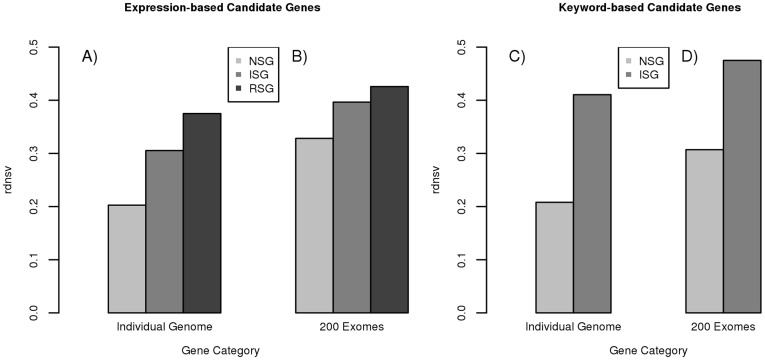
Relative density of nsSNVs (*rdnsv*) in different gene sets as estimated with different SNV datasets. Nervous system genes (NSG, light grey) show a smaller *rdnsv* than immune system genes (ISG, medium grey) or randomly sampled genes (RSG, dark grey) in a European diploid genome sequence (A, C) and a pooled set of 200 European exome sequences (B, D). The greater *rdnsv* in the pooled 200 exomes than the individual genome indicates an enrichment of nsSNVs among rare SNVs.

However, to additionally relate the *Rn*/*Rs* ratio to the neutral expectation, it is important to consider that transition mutations occur with higher likelihood than transversion mutations and that transitions are enriched among synonymous changes in the genetic code [31]. Here we correct for this nonsynonymous to synonymous mutation rate bias by multiplying the observed *Rn*/*Rs* ratio with a respective factor *f* that is defined by equation (2) in the Materials and Methods. This strategy is designed to estimate the relative density of nonsynonymous variants as compared with neutral expectation (*rdnsv*) as defined above by equation (3). We estimate *rdnsv* to be around 20% in NSGs, around 31% in ISGs and around 38% in RSGs with the SNVs from the diploid genome (Figure 1a, Table 1a). We next retrieve a second set of candidate genes through keyword search of the EntrezGene database [27]. These keyword-based candidates may differ from the expression-based candidates in the sense that they are more likely to have been experimentally studied in detail. When analyzing the SNVs from the diploid genome in these keyword-based candidates, the estimates of *rdnsv* are ∼21% in NSGs and ∼41% in ISGs (Figure 1c, Table 1a). Thus, also keyword-based NSGs again display a significantly (P<10^−5^) smaller level of nonsynonymous variation than ISGs (Table S2b).

**Table 1:** Relative density of nonsynonymous variants (*rdnsv*).

	RSG	expression ISG	expression NSG	keyword ISG	keyword NSG
**#genes**	1496	1496	1496	1040	1523
**A**)					
***rdnsv*** ** (diploid genome)**	0.38 [0.31–0.39]	0.31	0.20	0.41	0.21
***rdnsv*** ** (200 exomes)**	0.42 [0.39–0.45]	0.39	0.33	0.47	0.31
**B**)					
***rdnsv0*** ** (200 exomes)**	0.58 [0.53–0.65]	0.58	0.45	0.58	0.43
***rdnsv1*** ** (200 exomes)**	0.25 [0.21–0.29]	0.21	0.17	0.37	0.15

Candidate genes for the nervous system (NSG) and the immune system (ISG) are defined by tissue specific expression or keyword search and further compared with a set of randomly sampled genes (RSG). A) Overall *rdnsv* estimates for a diploid genome and 200 exome sequences, which reflect the density of nonsynonymous variants on a mixture of SNVs that range from rare to common in their population frequency. B) SNVs from the 200 exome dataset are additionally stratified by their derived allele frequency and a regression model is fitted to the values of *rdnsv*. The predicted value for the allele frequency of 0 is referred to as *rdnsv_0_*, whereas the predicted value for the allele frequency of 1 is referred to as *rdnsv_1_*. The interval in brackets shows the 2.5% and 97.5% quantiles from 10.000 random draws of genes.

**Figure 2:**
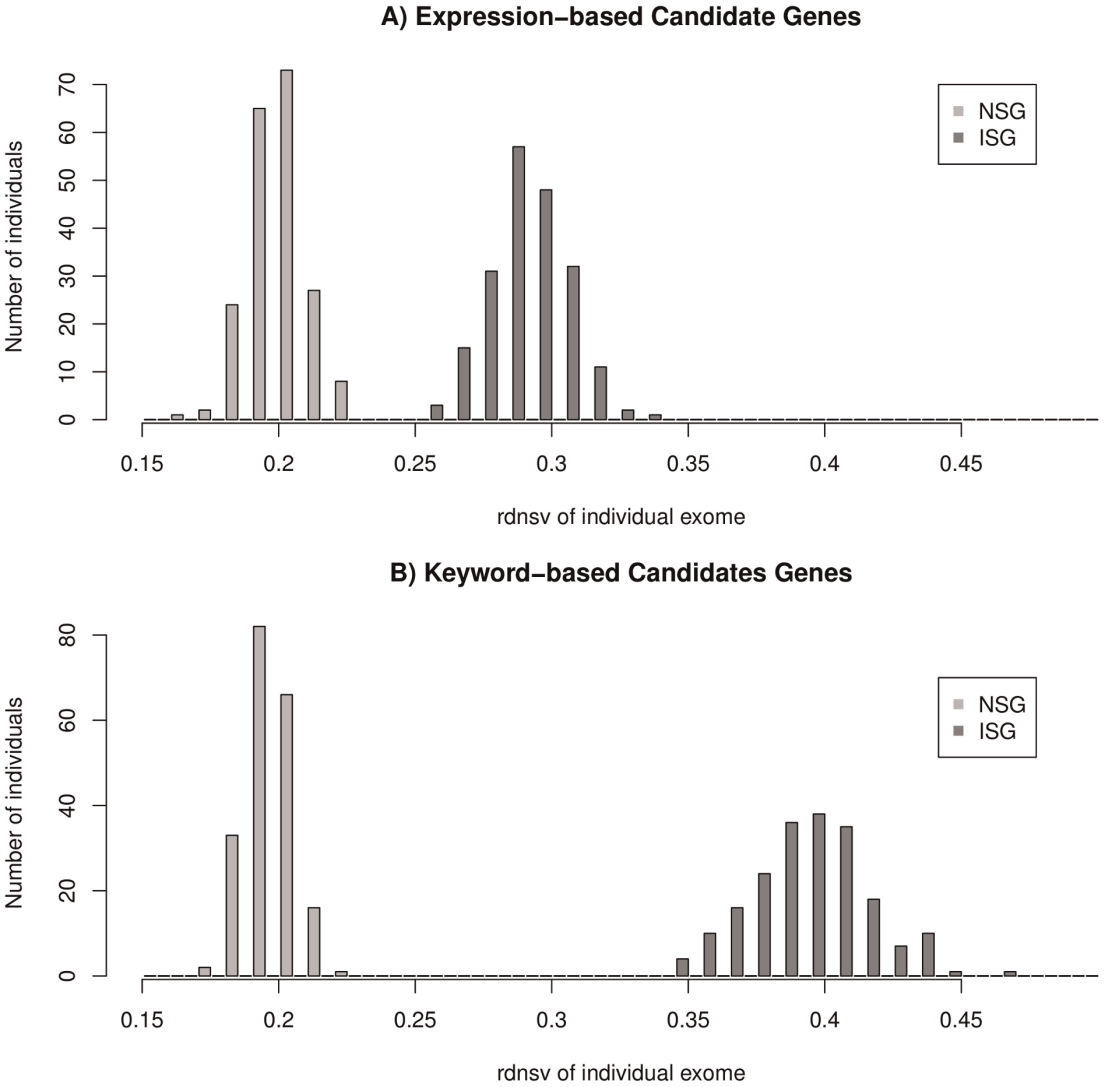
Distribution of *rdnsv* estimates over 200 individual exomes. A) expression-based candidate genes and B) keyword-based candidate genes. The value of *rdnsv* is estimated separately for each of the 200 exomes and found consistently smaller for NSGs (light grey) are than ISGs (medium grey). In addition, smaller estimates of *rdnsv* for expression-based ISGs than keyword-based ISGs are seen. No difference exists between expression-based NSGs and keyword-based NSGs.

To further expand these observation into a larger SNV dataset, we use a published dataset of 200 human exomes [12]. We first separately calculate *rdnsv* for each of the individual exomes, which shows *rdnsv* to be roughly normally distributed. The estimates of *rdnsv* are consistently smaller for NSGs than ISGs (Figure 2). The mean values of the distributions of *rdnsv* over the 200 individual exomes (20.1% and 29.0% for expression-based NSGs and ISGs and 19.8% and 39.3% for keyword-based NSGs and ISGs) are close to the corresponding *rdnsv* values from the diploid genome above, despite the fact the diploid genome was obtained under a rather different experimental protocol. Consistent with the diploid genome, we see a greater heterogeneity between expression- and keyword-based ISGs than the two types of NSGs.

It is important to note that the sampling of 400 instead of 2 chromosomes at each site causes an ascertainment of more rare SNVs. Because nsSNVs are enriched among rare SNVs, the estimates of *rdnsv* in the pooled 200 exomes are greater than those for the diploid genome in all three gene categories (Figure 1). Across the pooled dataset of 200 exomes, we estimate for expression-based candidates that *rdnsv* is ∼33% in NSGs, ∼39% in expression-based ISGs and ∼42% in RSGs, whereas for expression-based candidates it is 31% for NSGs and 47% for ISGs (Table 1a). Nevertheless, we again see that *rdnsv* is significantly smaller in NSGs than ISGs or RSGs (P<10^−3^), which applies to both expression-based and keyword-based candidates (Tables S2c, S2d).

To define the range of expected values of *rdnsv* for arbitrary sets of genes, we further randomly draw 10.000 sets of 1500 autosomal genes. This shows that both expression-based and keyword-based NSGs are at the low end of the range of *rdnsv* estimates (Table 1a). When comparing expression-based and keyword-based ISGs to each other, we see that the former tend to fall at the lower end of the range of *rdnsv* estimates, whereas the latter tend to fall on the upper end. Consistent with this greater heterogeneity of the two sets of ISGs than the two sets of NSGs, we see greater difference of the coding sequence length between the former than the latter two sets of genes (Figure S1b).

### Relative Density of nsSNVs as Stratified by Population Allele Frequency

**Figure 3:**
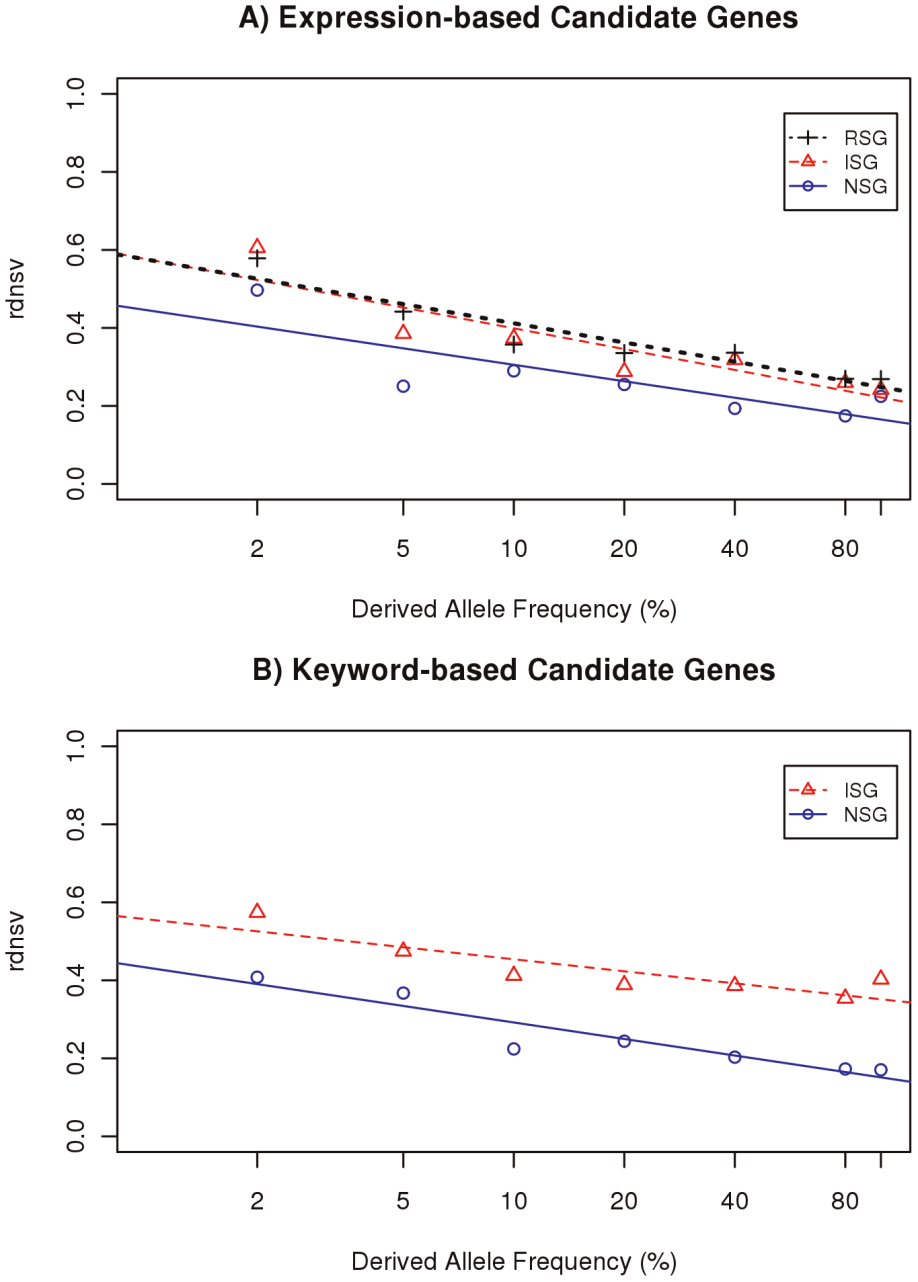
Estimates of *rdnsv* over different allele frequency bins. The estimates of *rdnsv* decrease with SNV allele frequency in all gene categories. The slope of the fitted regression model can be interpreted as a measure for the influence of purifying selection on segregating nsSNVs. The y-intercept (*rdnsv_0_*) can be interpreted as the proportion of nsSites where mutations are tolerated to segregate with an allele frequency notably greater than 0. A) Expression-based NSGs (circles), ISGs (triangles) or RSGs (crosses). The fitted models are *rdnsv*(NSG) = 0.45−0.061×; *rdnsv*(ISG) = 0.58−0.079× and *rdnsv*(RSG) = 0.58−0.071×B) Keyword based NSGs (blue) and ISGs (red). The fitted models are *rdnsv*(NSG) = 0.43−0.061×; *rdnsv*(ISG) = 0.58−0.045×.

When *rdnsv* is estimated in a diploid genome or a pooled set of chromosomes, its value reflects the level of nonsynonymous variation on a mixture of SNVs that range from rare to common in their population frequency. To additionally exploit the information that is contained in the change of *rdnsv* with allele frequency, we next group SNVs into disjoint frequency bins and separately estimate for each bin its *rdnsv* value in the pooled set of 200 exomes. This shows that expression-based NSGs display a reduced *rdnsv* value across all frequency bins (Figure 3a). When we further estimate *rdnsv* for our keyword-based candidate genes, we again see smaller *rdnsv* values for NSGs across all bins (Figure 3b). Additionally, *rdnsv* tends to decrease with SNV allele frequency in all sets of candidate genes.

To further capture this influence of allele frequency on *rdnsv*, we next fit a regression model to the estimates of *rdnsv* over the allele frequency of underlying SNVs in the 200 exome data. The y-intercept parameter (*rdnsv_0_*) of the model might be best interpreted as the proportion of nsSites where variants are tolerated to segregate with frequency greater 0 and thus do not cause any highly detrimental consequences. Analogously, we might interpret the predicted value for a derived allele frequency near 1 (*rdnsv_1_*) as the proportion of nsSites where mutations are not prevented from reaching fixation. In our candidate genes, we see that *rdnsv_0_* and *rdnsv_1_* equal 45% and 17% for expression-based NSGs, whereas they equal 58% and 21% for expression-based ISGs (Table 1b). For keyword-based NSGs we find that *rdnsv_0_* and *rdnsv_1_* equal 43% and 15%, whereas they equal 58% and 37% for keyword-based ISGs. Thus, both *rdnsv_0_* and *rdnsv_1_* are highly similar between expression- and keyword-based NSGs. In contrast, the two sets of ISGs display only similar estimates of *rdnsv_0_*, but quite different estimates of *rdnsv_1_*. The inspection of the fitted models shows that the y-intercept is significantly different from its theoretically expected value of 1 (P<0.001) and that the slope is significantly different from 0 (P<0.01) in all candidate sets. The coefficient of determination *R^2^* varies from 0.61 (expression-based NSGs) over 0.70 (keyword-based ISGs) and 0.78 (expression-based ISGs) to 0.85 (keyword-based NSGs), indicating that the models capture the dependency of *rdnsv* on SNV allele frequency. However, it can also be seen that the residual deviation from the fitted models consistently attains a relatively large value for the lowest frequency bin, which might indicate some non-linearity.

To relate the observed *rdnsv_0_* and *rdnsv_1_* of our candidate genes to their expected values, we further compare them with 10.000 draws of random genes sets (Table 1b). For expression-based and keyword-based NSGs we see that *rdnsv_0_* and as a consequence also *rdnsv_1_* fall at the low end of values. On the other hand, *rdnsv_1_* of expression-based ISGs tends to fall towards the lower end, whereas *rdnsv_1_* of keyword-based ISGs falls towards the upper end of values. Notably, both these sets of ISGs display *rdnsv_0_* similar to random gene sets. Thus, NSGs consistently stand out by their small *rdnsv_0_*, whereas the noted heterogeneity between expression-based and keyword-based ISGs becomes visible in a different slope, but not a different intercept of the two regression models.

We finally sought to find out how *rdnsv* estimates of our broadly defined candidate genes compare to the *rdnsv* of gene sets with more specific molecular annotations. To address this question we retrieved all sets of gene ontology (GO) annotated genes that harbor at least 1000 coding SNVs in the 200 exome dataset. We then estimate for each GO-category its mean value of *rdnsv* across the 200 exomes as well as its values of *rdnsv_0_* and *rdnsv_1_* from the respective regression models over the allele frequency strata (Table S3). This shows several GO-categories for nervous system functions (e.g. GO:0048812, GO:0007409, GO:0045202) to belong to those with the smallest level of nonsynonymous variation (Table 2a). Consistent with the above results for expression-based and keyword-based NSGs, see that *rdnsv_0_* is reduced in nervous system genes. In addition, GO-categories related to tyrosine kinase signaling (GO:0007169, GO:0007167) display the lowest levels of nonsynonymous variation. It remains to be found out, whether this reduction constitutes a feature that is genuine to tyrosine kinase signaling genes or whether it is driven by their functions in nervous system cells.

**Table 2:** Estimates of *rdnsv* in the 200 exomes in sets of genes as defined by ontology (GO) annotations.

Gene Ontology (GO) category	genes	nsSNVs	sSNVs	mean rdnsv	sd rdnsv	rdnsv0	rdnsv1
**A**)							
GO:0007169:transmembrane_receptor_tyrosine_kinase_signaling	313	365	636	0.121	0.011	0.385	0.072
GO:0007167:enzyme_linked_receptor_protein_signaling_pathway	394	466	771	0.128	0.01	0.397	0.095
GO:0006935:chemotaxis	337	405	669	0.136	0.01	0.401	0.094
GO:0048812:neuron_projection_morphogenesis	326	445	703	0.139	0.011	0.396	0.11
GO:0007409:axonogenesis	303	420	664	0.141	0.012	0.397	0.11
GO:0031175:neuron_projection_development	368	510	795	0.143	0.011	0.4	0.119
GO:0048667:cell_morphogenesis_involved_in_neuron_differentiation	322	446	694	0.146	0.012	0.397	0.12
GO:0043005:neuron_projection	411	587	831	0.15	0.01	0.484	0.1
GO:0045202:synapse	326	477	633	0.151	0.013	0.544	0.089
GO:0048666:neuron_development	436	578	890	0.153	0.011	0.399	0.126
**B)**							
GO:0005815:microtubule_organizing_center	277	542	484	0.344	0.025	0.5	0.399
GO:0005576:extracellular_region	1322	2281	1948	0.345	0.012	0.644	0.341
GO:0006952:defense_response	525	764	691	0.351	0.022	0.556	0.345
GO:0006955:immune_response	500	696	625	0.357	0.023	0.533	0.393
GO:0004871:signal_transducer_activity	1170	2350	1994	0.4	0.016	0.586	0.369
GO:0004872:receptor_activity	1233	2710	2218	0.406	0.015	0.615	0.368
GO:0038023:signaling_receptor_activity	913	2046	1583	0.456	0.019	0.624	0.43
GO:0004888:transmembrane_signaling_receptor_activity	844	1991	1489	0.466	0.02	0.654	0.437
GO:0004930:G-protein_coupled_receptor_activity	604	1469	890	0.603	0.029	0.741	0.573
GO:0004984:olfactory_receptor_activity	310	1024	430	0.896	0.048	1.011	0.993

The 10 GO-categories with the smallest (A) and the greatest (B) mean values of *rdnsv* are shown. The full list of all GO-categories with at least 1000 coding SNVs is given in Table S3. For each category the number of annotated genes, nonsynonymous and synonymous SNVs, the mean and standard deviation of individual *rdnsv* estimates across the 200 exomes, as well as the values of *rdnsv_0_* and *rdnsv_1_*, are shown.

On the other end of the spectrum, we see the highest level of nonsynonymous variation for GO-categories related to olfactory receptor function (GO:0004984, GO:0004930) as well as immune system function (GO:0006955, GO:0006952) (Table 2b). GO ‘olfactory receptor’ genes display both *rdnsv_0_* and *rdnsv_1_* values fairly close to 1, i.e. both *rdnsv_0_* and *rdnsv_1_* account for the increased overall *rdnsv* in individual exomes. In contrast, GO ‘immune response’ genes show *rdnsv_0_* values roughly equal to random gene sets, but greater *rdnsv_1_* values, which is similar as seen for keyword-based ISGs. These observations suggests that the high overall *rdnsv* of ‘olfactory receptor’ genes is largely due to relaxed constraint, whereas the high overall *rdnsv* of ‘immune response’ genes might be due to an influence of positive selection.

## Discussion

We devise a method for estimating the relative density of nonsynonymous variants as compared with the neutral expectation (*rdnsv*), which we apply to two separate exome datasets [12], [13]. We notice that *rdnsv* shows relatively small differences between individual exomes, but strong difference between gene categories. To capture the dependency of *rdnsv* on the allele frequency of SNVs, we fit a regression model to the values of *rdnsv* as stratified by the frequency of SNVs. We interpret the slope of this model as a measure of the overall strength of selection on segregating nsSNVs. The y-intercept of this model (*rdnsv_0_*) may be interpreted as a prediction of the proportion of nsSites (among all nsSites) where mutations are tolerated to segregate in the population.

We use our method to measure the levels of nonsynonymous variants (nsSNVs) among human nervous system genes (NSGs), immune system genes (ISGs), randomly sampled genes (RSGs) and GO-annotated genes. That *rdnsv* is consistently reduced for NSGs is indicating stronger purifying selection. We find that the reduced overall *rdnsv* values of NSGs are paralleled by smaller estimates of *rdnsv_0_*. This smaller *rdnsv_0_* predicts greater proportions of nsSites that are intolerant to mutations as an important cause for the reduced overall *rdnsv*. Such a prediction of a greater proportion of strongly deleterious nsSites in NSGs is consistent with the high frequency of neurological symptoms among undiagnosed disease phenotypes [32] as well as the greater number of established monogenic disease genes in the OMIM database. Based on an analysis of the OMIM database [33], about 2.4 as many monogenic disease genes for the nervous system than the immune system have been discovered (326 to 135). In contrast, about 3.8 as many susceptibility loci were identified for immune phenotypes than nervous system phenotypes (122 to 32), based on the NHGRI GWAS catalog [34] (Figure S2). Our present study demonstrates how these different rates of monogenic disease manifestations for different phenotypes are reflected in the sequence variability patterns of a population-based sample. The reduced level of nonsynonymous variation in NSGs is also consistent with the strong conservation of such genes among mammals [15], [35]. The presented analysis additionally suggests that a greater proportion of mutation intolerant sites has made a major contribution to the increased interspecies conservation. Stronger purifying selection on nervous system genes could be caused by the functional complexity of neuronal cells and the developmental complexity of the nervous system. A larger proportion of highly deleterious nsSites in NSGs is further consistent with the role of exomic *de-novo* mutations in mental retardation [36], schizophrenia [37], and autism [38]–[40].

On the other hand, it has been hypothesized that functional variants in immune genes are more often positively selected due to pressures that were imposed by infectious agents [15], [41]–[44]. Here we see that *rdnsv* values are increased for certain sets of ISGs (i.e. keyword-based ISGs and GO-immune response genes). Importantly, any influence of positive selection would not be expected to influence the intercept (*rdnsv_0_*) of the regression models, because it would alter the allele frequencies of segregating SNVs and not the proportion of nsSites that tolerate mutations. Instead, the slope of the model reflects the overall strength of selection on segregating nsSNVs, which influences the proportion of nsSites where nsSNVs may become fixed (*rdnsv_1_*). Therefore, it is interesting that the greater overall *rdnsv* for certain types of ISGs in individual exomes is paralleled by an increased estimate of *rdnsv_1_*, but not an increased *rdnsv_0_*. This seems to support positive selection over relaxed constraint as possible explanation. Although more prevalent positive selection in ISGs may contribute to the presence of more common nsSNVs and putative disease variants, it nevertheless needs to be pointed out that all tested sets of immunological genes still display a negative slope parameter. This is consistent with a general importance of purifying selection for the frequency spectrum of segregating nsSNVs in ISGs too. It remains to be found out in future studies, which molecular subsets of ISGs are more likely to harbour SNVs that are influenced by positive selection and whether the observed heterogeneity might be due to differences between genes that function in the innate and the adaptive immune system.

It is important to distinguish our approach for analyzing the level of nonsynonymous variation from methods for calculating the *Ka/Ks* ratio that are widely employed in comparative genomic studies [45]. In particular, one may want to compare our normalization strategy to approximate methods for calculating the *Ka/Ks* ratio under a two parameter model with different rates of transitions and transversions [46], [47]. However, our method is designed to evaluate variants from individual human genome sequences, whereas *Ka/Ks* ratio methods are designed to analyze fixed differences between sequences that are millions of years apart and connected by a phylogenetic tree. From a technical point of view, different mutational pathways between codons and recurrent mutation of a same site have to be considered for comparative genomic data, but not for human SNV data. On the other hand, no consistent phylogenetic tree exists for a set of individual human genome sequences. Therefore, a different set of methods has to be used to analyze SNV data.

To our knowledge, the proposed approach has not been used before to analyze individual genome data. Our estimates of the proportions of nsSites that are tolerant and intolerant to mutations are consistent with earlier studies that estimated the proportions of neutral, weakly and strongly deleterious nsSites in human genes by different methods [9], [12], [48] and from human and chimp comparison [15]. In addition, the confidence in our normalization procedure may be strengthened by the estimates of *rdnsv* close to 1 for olfactory receptor activity genes, because those genes are known to be degenerating in the human lineage and evolve largely neutral [49]. Nevertheless, it may be kept in mind that some uncertainty is introduced by the assumption that sSNVs evolve neutrally, which might not always be the case due to selection on synonymous mutations [50] or background selection [51]. Also it might be possible to refine the method by taking hypermutability of CpG sites into account [52]. However, these factors do not alter the comparison of sets of genes, as long as they influence nsSites and sSites homogeneously across categories. Furthermore, one might try to advance the approach by fitting more complex statistical models than a regression line over the frequency stratified *rdnsv* estimates. In this context, it is noteworthy that the *rdnsv* estimate for the lowest allele frequency consistently shows a relatively large positive deviation from the linear model across the analyzed genes sets. This could indicate a non-linearity that might be better captured by a more complex model that may be fitted to larger SNV datasets becoming available in future. However, it might also be influenced by a higher false positive SNV call rate in the lowest frequency bin, what may also be considered by subsequent modeling approaches.

In conclusion, we propose a novel statistical method that estimates the relative density of nonsynonymous variants (*rdnsv*) in a set of human genes. We are convinced that in many situations any possible sources of impreciseness are outweighed by the advantage of its practical simplicity (in the simplest form, multiplying an observed ratio between nsSNVs and sSNVs by f’ (∼0.4) and plotting this transformed ratio over the allele frequency). Using this method we explain here, why the nervous system is more often affected by monogenic diseases than the immune system. We would expect that the described method will turn out to be useful for other questions too.

## Supporting Information

Figure S1 **Mean number of coding nucleotide sites per gene for different sets of candidate genes.** a) Expression-based candidates and b) keyword-based candidates. Sites are defined as mutational opportunities in the reference sequence. Sites are stratified as nonsynonymous (dark grey) and synonymous (light grey). The mean number of nonsynonymous and synonymous sites is greater in nervous system genes than immune system genes or random genes. Error bars denote two standard errors of the mean.
(PDF)

Figure S2 **Number of monogenic disease genes from the OMIM database and complex disease loci from the GWAS-catalog (both queried 03/10).** Nervous system phenotypes are more often linked to monogenic disease genes and therefore have more entries in OMIM (dark grey bars). Vice versa, immune system phenotypes are more often linked to complex susceptibility loci and therefore have more entries in the GWAS-catalog (light grey bars).
(PDF)

Table S1 **Number of synonymous and nonsynonymous sites in different sets of genes in the human genome reference sequence.** Sites are defined as mutational opportunities in the coding sequence of genes. Sites may be classified synonymous (*sSite*) or nonsynonymous (*nsSite*). In a similar sense, each site can be a transition site or transversion site. Among sSites, the ratio of transition and transversion sites (*sSite_ts_*/*sSite_tv_*) is close to 1, whereas among nsSites this ratio (*nsSite_ts_*/*nsSite_tv_*) is close to 0.4. Candidate genes for the nervous system (NSG) and the immune system (ISG) are defined by tissue specific expression and keyword search and further compared with randomly sampled genes (RSG).
(PDF)

Table S2 **Number of** **SNVs in different sets of candidate genes in the diploid genome and 200 exomes.** For each gene set, its total number of nsSNVs and sSNVs, its rate of *nsSNVs/nsSite* (*Rn*) and its rate *sSNVs/sSite* (*Rs*) are shown. Immune System Genes (ISG) and a set of randomly sampled genes (RSG) are compared to nervous system genes (NSG) based on 10,000 permutation of gene category labels. The intervals for RSGs in square brackets show the 2.5% and 97.5% quantiles from 10.000 random draws of genes. To further estimate the relative density of nsSNVs (*rdnsv*) from the *Rn/Rs* ratio, the respective correction factor *f* needs to be applied, which is described in the main text.
(PDF)

Table S3 **Estimates of** ***rdnsv*** **in the 200 exomes in sets of genes as defined by ontology (GO) annotations.** For each category the number of annotated genes, nonsynonymous and synonymous SNVs, the mean and standard deviation of individual *rdnsv* estimates across the 200 exomes, as well as the values of *rdnsv_0_* and *rdnsv_1_*, are shown.
(PDF)
