# Supplementary figures and images for: A Simple Method for Analyzing Exome Sequencing Data Shows Distinct Levels of Nonsynonymous Variation for Human Immune and Nervous System Genes
Source: PLoS One. 2012 Jun 6;7(6):e38087. doi: 10.1371/journal.pone.0038087 (PMC3368947; doi:10.1371/journal.pone.0038087)

A) Expression-based candidates

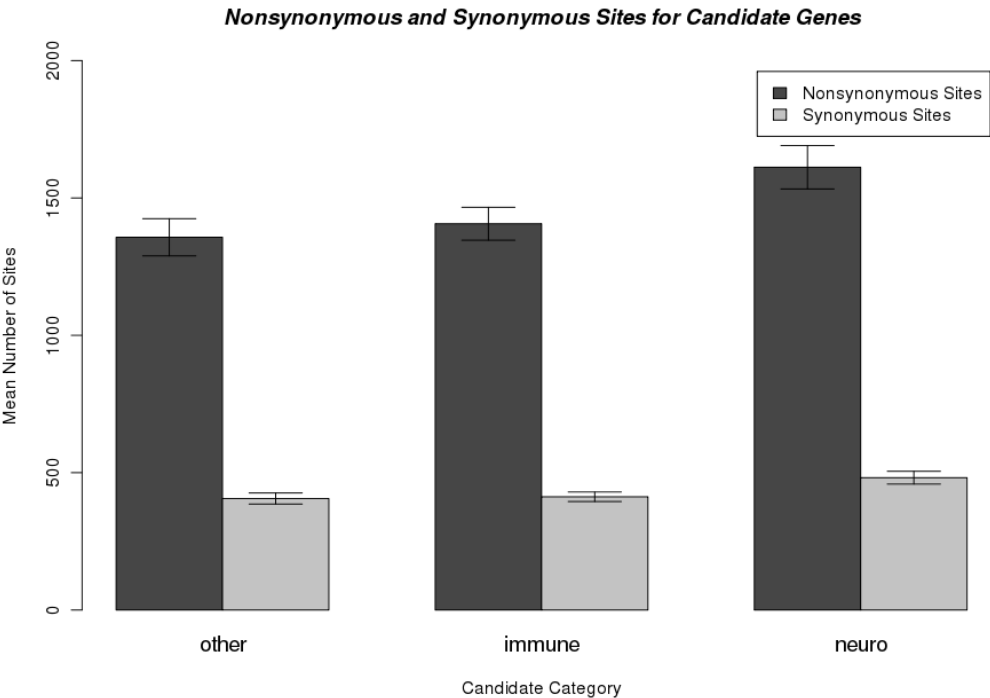

B) Keyword-based candidates

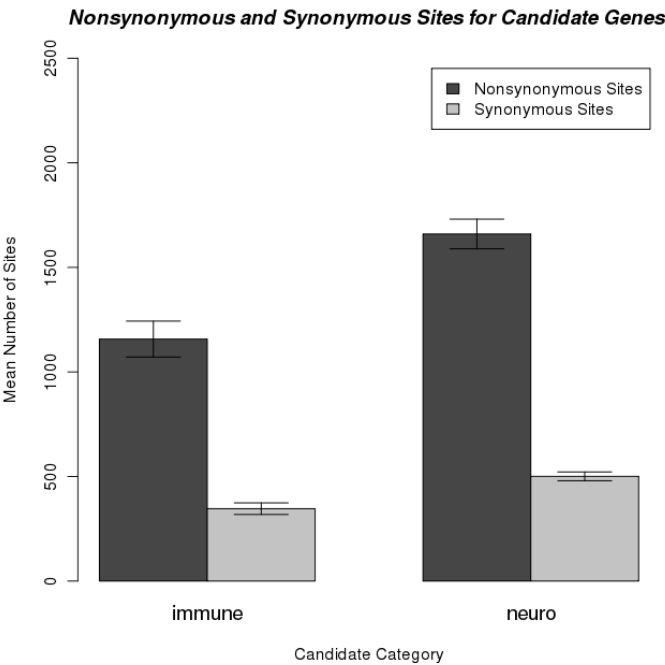

Supplement: Figure S1 — Mean number of coding nucleotide sites per gene for different sets of candidate genes. a) Expression-based candidates and b) keyword-based candidates. Sites are defined as mutational opportunities in the reference sequence. Sites are stratified as nonsynonymous (dark grey) and synonymous (light grey). The mean number of nonsynonymous and synonymous sites is greater in nervous system genes than immune system genes or random genes. Error bars denote two standard errors of the mean. (PDF) [file pone.0038087.s001.pdf]

**Disease Gene Loci Recorded in OMIM and the GWAS catalog**

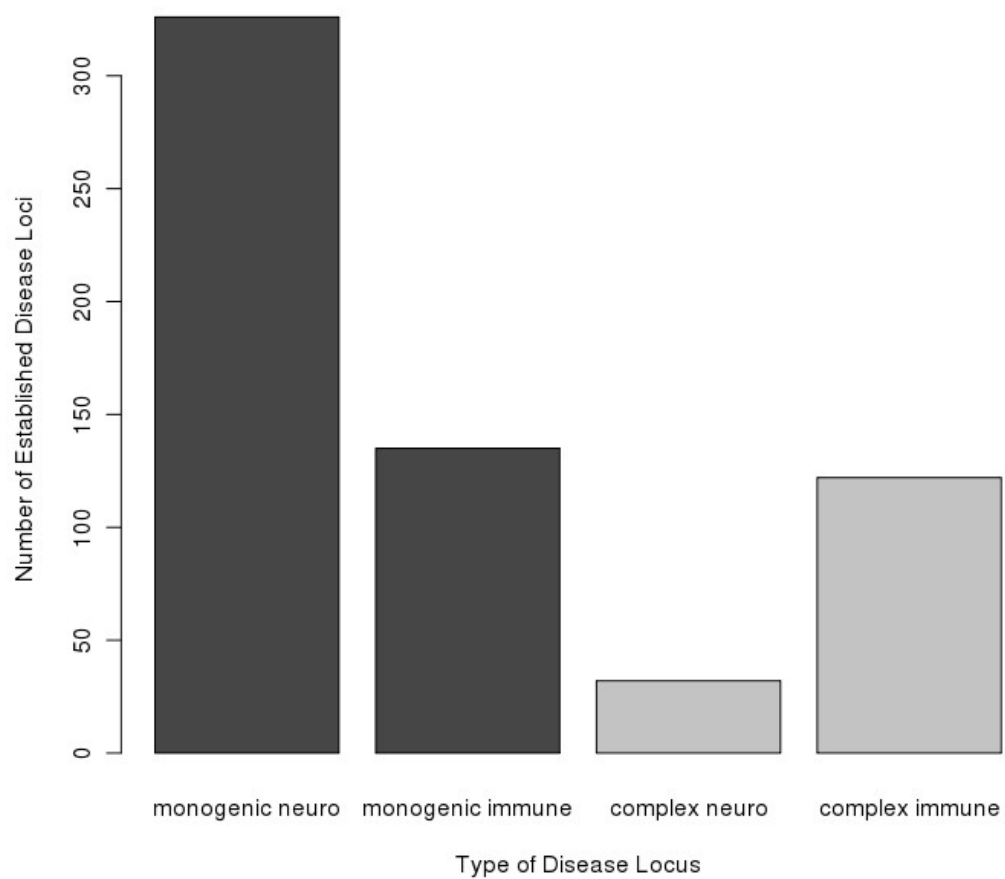

Supplement: Figure S2 — Number of monogenic disease genes from the OMIM database and complex disease loci from the GWAS-catalog (both queried 03/10). Nervous system phenotypes are more often linked to monogenic disease genes and therefore have more entries in OMIM (dark grey bars). Vice versa, immune system phenotypes are more often linked to complex susceptibility loci and therefore have more entries in the GWAS-catalog (light grey bars). (PDF) [file pone.0038087.s002.pdf]
