# Supplementary material for: A Simple Method for Analyzing Exome Sequencing Data Shows Distinct Levels of Nonsynonymous Variation for Human Immune and Nervous System Genes
Source: PLoS One. 2012 Jun 6;7(6):e38087. doi: 10.1371/journal.pone.0038087 (PMC3368947; doi:10.1371/journal.pone.0038087)
Supplement: Table S1 — Number of synonymous and nonsynonymous sites in different sets of genes in the human genome reference sequence. Sites are defined as mutational opportunities in the coding sequence of genes. Sites may be classified synonymous (sSite) or nonsynonymous (nsSite). In a similar sense, each site can be a transition site or transversion site. Among sSites, the ratio of transition and transversion sites (sSitets/sSitetv) is close to 1, whereas among nsSites this ratio (nsSitets/nsSitetv) is close to 0.4. Candidate genes for the nervous system (NSG) and the immune system (ISG) are defined by tissue specific expression and keyword search and further compared with randomly sampled genes (RSG). (PDF) [file pone.0038087.s003.pdf]

Number of synonymous and nonsynonymous sites in the human reference sequence.

**candidate status**	***sSite* / 10E03**	$sSite_{ts}$ / $sSite_{tv}$	$sSite_{ts}$ / $sSite$	***nsSite* / 10E03**	$nsSite_{ts}$ / $nsSite_{tv}$	$nsSite_{ts}$ / $nsSite$
**RSG**	608.7	0.982	0.495	2035.4	0.398	0.285
**expression-based ISG**	616.7	1	0.5	2103.5	0.395	0.283
**expression-based NSG**	720.6	0.98	0.495	2411.1	0.398	0.285
**keyword-based ISG**	359.9	0.98	0.495	1203.3	0.399	0.285
**keyword-based NSG**	762.5	0.978	0.494	2527.9	0.4	0.286
