# Supplementary material for: A Simple Method for Analyzing Exome Sequencing Data Shows Distinct Levels of Nonsynonymous Variation for Human Immune and Nervous System Genes
Source: PLoS One. 2012 Jun 6;7(6):e38087. doi: 10.1371/journal.pone.0038087 (PMC3368947; doi:10.1371/journal.pone.0038087)
Supplement: Table S2 — Number of SNVs in different sets of candidate genes in the diploid genome and 200 exomes. For each gene set, its total number of nsSNVs and sSNVs, its rate of nsSNVs/nsSite (Rn) and its rate sSNVs/sSite (Rs) are shown. Immune System Genes (ISG) and a set of randomly sampled genes (RSG) are compared to nervous system genes (NSG) based on 10,000 permutation of gene category labels. The intervals for RSGs in square brackets show the 2.5% and 97.5% quantiles from 10.000 random draws of genes. To further estimate the relative density of nsSNVs (rdnsv) from the Rn/Rs ratio, the respective correction factor f needs to be applied, which is described in the main text. (PDF) [file pone.0038087.s004.pdf]

A) coding SNVs from the diploid genome in expression-based candidates								
**candidate status**	**nsSNVs (miss, nons)**	**Rn / 10E-03**	**comparison to NSG by permutation**	**sSNVs**	**Rs / 10E-03**	**comparison to NSG by permutation**	**Rn / Rs**	**comparison to NSG**
**RSG**	743 (737,6)	0.365 [0.30-0.38]	P < 1E-5	800	1.31 [1.19-1.41]	P= 0.08418	0.278	P < 1E-05
**ISG**	571 (568,3)	0.271	P < 1E-5	740	1.199	P= 0.78416	0.226	P= 10E-5
**NSG**	427 (424,3)	0.177	NA	850	1.179	NA	0.15	NA

B) coding SNVs from the diploid genome in keyword-based candidates								
**candidate status**	**nsSNVs (miss, nons)**	**Rn / 10E-03**	**comparison to NSG by permutation**	**sSNVs**	**Rs / 10-E3**	**comparison to NSG by permutation**	**Rn / Rs**	**comparison to NSG by permutation**
**ISG**	578	0.4803	P < 1E-05	570	1.413	P = 0.02393	0.303	P < 1E-05
**NSG**	571	0.2258	NA	1078	1.583	NA	0.159	NA

C) coding SNVs from the 200 exomes in expression-based candidates								
**candidate status**	**nsSNVs (miss, nons)**	**Rn / 10E-03**	**comparison to NSG by permutatio n**	**sSNVs**	**Rs / 10E-03**	**comparison to NSG by permutation**	**Rn / Rs**	**comparison to NSG by permutation**
**RSG**	2324 (2290,34)	1.14 [1.03-1.19]	P < 1E-05	2204	3.62 [3.31-3.77]	P = 0.00073	0.31	P < 1E-05
**ISG**	2137 (2112,25)	1.01	P < 1E-05	2133	3.458	P = 0.01389	0.29	P = 0.00022
**NSG**	1807 (1791,16)	0.749	NA	2221	3.08	NA	0.243	NA

D) coding SNVs from the 200 exomes in keyword-based candidates								
**candidate status**	**nsSNVs (miss, nons)**	**Rn / 10E-03**	**comparison to NSG by permutation**	**sSNVs**	**Rs / 10E-03**	**comparison to NSG by permutation**	**Rn / Rs**	**comparison to NSG by permutation**
**ISG**	1708 (1684/24)	1.419	P < 1 E-05	1462	3.89	P = 0.28535	0.349	P < 1 E-05
**NSG**	2258 (2239/19)	0.8932	NA	2971	4.06	NA	0.229	NA
