# Supplementary material for: A Simple Method for Analyzing Exome Sequencing Data Shows Distinct Levels of Nonsynonymous Variation for Human Immune and Nervous System Genes
Source: PLoS One. 2012 Jun 6;7(6):e38087. doi: 10.1371/journal.pone.0038087 (PMC3368947; doi:10.1371/journal.pone.0038087)
Supplement: Table S3 — Estimates of rdnsv in the 200 exomes in sets of genes as defined by ontology (GO) annotations. For each category the number of annotated genes, nonsynonymous and synonymous SNVs, the mean and standard deviation of individual rdnsv estimates across the 200 exomes, as well as the values of rdnsv0 and rdnsv1, are shown. (PDF) [file pone.0038087.s005.pdf]

GO_Category	genes	nsSNVs	sSNVs	mean rdnsv	sd rdnsv	rdnsv0	rdnsv1
GO:0007169:transmembrane_receptor_tyrosine_kinase_signaling	313	365	636	0.120522	0.0113	0.385	0.072
GO:0007167:enzyme_linked_receptor_protein_signaling_pathway	394	466	771	0.127987	0.0102	0.397	0.095
GO:0006935:chemotaxis	337	405	669	0.136293	0.0104	0.401	0.094
GO:0048812:neuron_projection_morphogenesis	326	445	703	0.13896	0.0114	0.396	0.11
GO:0007409:axonogenesis	303	420	664	0.141014	0.0115	0.397	0.11
GO:0031175:neuron_projection_development	368	510	795	0.143313	0.0109	0.4	0.119
GO:0048667:cell_morphogenesis_involved_in_neuron_differentia	322	446	694	0.145977	0.0118	0.397	0.12
GO:0043005:neuron_projection	411	587	831	0.149634	0.0104	0.484	0.1
GO:0045202:synapse	326	477	633	0.151173	0.0134	0.544	0.089
GO:0048666:neuron_development	436	578	890	0.152631	0.0107	0.399	0.126
GO:0030182:neuron_differentiation	516	667	999	0.153033	0.0098	0.423	0.127
GO:0000904:cell_morphogenesis_involved_in_differentiation	367	543	791	0.155903	0.0113	0.433	0.125
GO:0007268:synaptic_transmission	367	456	698	0.157652	0.0121	0.399	0.155
GO:0048699:generation_of_neurons	664	863	1266	0.161579	0.0083	0.417	0.149
GO:0019226:transmission_of_nerve_impulse	411	503	759	0.163673	0.012	0.394	0.166
GO:0035637:multicellular_organismal_signaling	411	503	759	0.163673	0.012	0.394	0.166
GO:0040011:locomotion	605	872	1180	0.165124	0.0091	0.483	0.125
GO:0030054:cell_junction	412	704	876	0.166789	0.0123	0.577	0.101
GO:0060589:nucleoside-triphosphatase_regulator_activity	325	553	730	0.166863	0.0129	0.499	0.118
GO:0009605:response_to_external_stimulus	703	972	1288	0.167815	0.0091	0.52	0.117
GO:0030695:GTPase_regulator_activity	317	552	723	0.16895	0.013	0.502	0.119
GO:0007267:cell-cell_signaling	568	638	909	0.169132	0.011	0.443	0.147
GO:0022008:neurogenesis	702	948	1346	0.169413	0.0081	0.429	0.154
GO:0001775:cell_activation	350	482	627	0.170157	0.0131	0.51	0.125
GO:0016773:phosphotransferase_activity,_alcohol_group_as_acc	577	842	1121	0.173484	0.0112	0.467	0.139
GO:0009719:response_to_endogenous_stimulus	569	692	913	0.175052	0.0116	0.471	0.144
GO:0051056:regulation_of_small_GTPase_mediated_signal_tran	249	479	609	0.176382	0.0158	0.504	0.118
GO:0045859:regulation_of_protein_kinase_activity	360	471	624	0.177153	0.0139	0.499	0.133
GO:0016310:phosphorylation	481	588	837	0.178544	0.0128	0.392	0.156
GO:0048468:cell_development	737	1010	1340	0.178923	0.0098	0.466	0.14
GO:0030001:metal_ion_transport	323	460	634	0.179418	0.014	0.484	0.134
GO:0031399:regulation_of_protein_modification_process	578	715	918	0.18	0.0122	0.493	0.137
GO:0043549:regulation_of_kinase_activity	381	515	676	0.181032	0.0141	0.497	0.139
GO:0001932:regulation_of_protein_phosphorylation	466	585	753	0.181724	0.0134	0.511	0.136
GO:0016477:cell_migration	340	499	633	0.182463	0.0146	0.504	0.154
GO:0004672:protein_kinase_activity	483	722	949	0.183206	0.0127	0.465	0.146
GO:0010646:regulation_of_cell_communication	768	957	1282	0.183792	0.0107	0.453	0.154
GO:0048858:cell_projection_morphogenesis	375	595	790	0.184192	0.0118	0.448	0.161
GO:0032268:regulation_of_cellular_protein_metabolic_process	709	810	1072	0.184493	0.0111	0.452	0.146
GO:0051338:regulation_of_transferase_activity	390	524	679	0.18504	0.0142	0.502	0.143
GO:0010647:positive_regulation_of_cell_communication	379	482	644	0.185201	0.0152	0.451	0.144
GO:0032990:cell_part_morphogenesis	379	597	791	0.18557	0.0118	0.448	0.162
GO:0023056:positive_regulation_of_signaling	381	486	650	0.185964	0.0156	0.456	0.142
GO:0016301:kinase_activity	652	984	1249	0.186251	0.0111	0.488	0.147
GO:0007399:nervous_system_development	1083	1550	1988	0.186352	0.008	0.475	0.172
GO:0006812:cation_transport	390	564	730	0.186553	0.0135	0.507	0.147
GO:0003779:actin_binding	240	511	593	0.186623	0.0167	0.563	0.14

GO:0007420:brain_development	312	430	572	0.188126	0.0157	0.446	0.212
GO:0010942:positive_regulation_of_cell_death	363	510	624	0.188133	0.0143	0.526	0.171
GO:0043085:positive_regulation_of_catalytic_activity	552	703	926	0.188169	0.0129	0.462	0.151
GO:0008324:cation_transmembrane_transporter_activity	418	585	802	0.188688	0.0128	0.45	0.144
GO:0006915:apoptosis	467	587	776	0.188762	0.0137	0.449	0.146
GO:0070727:cellular_macromolecule_localization	390	522	630	0.189018	0.0149	0.501	0.112
GO:0010604:positive_regulation_of_macromolecule_metabolic_p	903	998	1350	0.18929	0.0114	0.424	0.147
GO:0012501:programmed_cell_death	477	595	788	0.189632	0.0137	0.444	0.146
GO:0042325:regulation_of_phosphorylation	497	648	825	0.189865	0.0134	0.498	0.149
GO:0051674:localization_of_cell	376	613	718	0.189947	0.0134	0.557	0.149
GO:0048870:cell_motility	376	613	718	0.189947	0.0134	0.557	0.149
GO:0019725:cellular_homeostasis	389	487	636	0.189994	0.0139	0.462	0.2
GO:0030030:cell_projection_organization	482	776	1004	0.190529	0.0112	0.453	0.167
GO:0051174:regulation_of_phosphorus_metabolic_process	513	665	842	0.190972	0.0137	0.504	0.151
GO:0019220:regulation_of_phosphate_metabolic_process	513	665	842	0.190972	0.0137	0.504	0.151
GO:0023057:negative_regulation_of_signaling	329	417	591	0.190974	0.015	0.409	0.163
GO:0072359:circulatory_system_development	392	561	775	0.191049	0.0143	0.425	0.162
GO:0072358:cardiovascular_system_development	392	561	775	0.191049	0.0143	0.425	0.162
GO:0010648:negative_regulation_of_cell_communication	329	418	591	0.191081	0.015	0.412	0.163
GO:0006468:protein_phosphorylation	401	511	716	0.1912	0.0133	0.388	0.17
GO:0042995:cell_projection	749	1418	1608	0.19135	0.0089	0.602	0.144
GO:0010557:positive_regulation_of_macromolecule_biosynthetic_	643	714	926	0.191482	0.014	0.453	0.152
GO:0044451:nucleoplasm_part	457	456	596	0.191537	0.0165	0.477	0.161
GO:0043065:positive_regulation_of_apoptosis	353	499	594	0.191548	0.0148	0.545	0.172
GO:0043068:positive_regulation_of_programmed_cell_death	354	500	596	0.191668	0.0147	0.545	0.172
GO:0008284:positive_regulation_of_cell_proliferation	388	427	586	0.191713	0.0168	0.412	0.134
GO:0034613:cellular_protein_localization	387	521	621	0.191797	0.0151	0.506	0.113
GO:0061024:membrane_organization	316	535	644	0.191893	0.0141	0.545	0.148
GO:0016044:cellular_membrane_organization	315	535	644	0.191893	0.0141	0.545	0.148
GO:0007417:central_nervous_system_development	428	591	764	0.192519	0.0133	0.468	0.194
GO:0016265:death	566	719	923	0.192707	0.0129	0.456	0.16
GO:0042127:regulation_of_cell_proliferation	686	803	1068	0.193078	0.0122	0.415	0.16
GO:0008219:cell_death	563	719	922	0.193437	0.0129	0.455	0.163
GO:0031325:positive_regulation_of_cellular_metabolic_process	934	1041	1353	0.193644	0.0116	0.439	0.151
GO:0045595:regulation_of_cell_differentiation	553	687	869	0.193818	0.0128	0.488	0.185
GO:0016772:transferase_activity,_transferring_phosphorus-conta	751	1155	1398	0.194148	0.0102	0.501	0.16
GO:0009967:positive_regulation_of_signal_transduction	356	468	594	0.194435	0.0163	0.48	0.15
GO:0007186:G-protein_coupled_receptor_signaling_pathway	385	508	675	0.19452	0.0142	0.456	0.141
GO:0000902:cell_morphogenesis	456	741	937	0.194902	0.0112	0.476	0.167
GO:0015075:ion_transmembrane_transporter_activity	547	853	1114	0.195177	0.0105	0.486	0.148
GO:0009725:response_to_hormone_stimulus	475	609	747	0.195188	0.014	0.493	0.171
GO:0009891:positive_regulation_of_biosynthetic_process	714	796	1022	0.195661	0.013	0.453	0.155
GO:0035556:intracellular_signal_transduction	933	1246	1539	0.196455	0.0093	0.509	0.154
GO:0005216:ion_channel_activity	313	506	690	0.196637	0.0126	0.455	0.139
GO:0009893:positive_regulation_of_metabolic_process	975	1108	1457	0.196734	0.0113	0.432	0.151
GO:0031324:negative_regulation_of_cellular_metabolic_process	653	670	890	0.197214	0.0137	0.403	0.169
GO:0048585:negative_regulation_of_response_to_stimulus	375	483	647	0.197368	0.0146	0.451	0.173
GO:0031328:positive_regulation_of_cellular_biosynthetic_process	706	794	1012	0.197489	0.0133	0.459	0.156
GO:0022891:substrate-specific_transmembrane_transporter_acti	632	954	1241	0.197576	0.0105	0.489	0.153

GO:0044092:negative_regulation_of_molecular_function	383	445	584	0.19871	0.0165	0.426	0.173
GO:0023051:regulation_of_signaling	1119	1584	1980	0.198962	0.0091	0.483	0.164
GO:0004674:protein_serine/threonine_kinase_activity	335	503	599	0.199132	0.0147	0.525	0.161
GO:0051246:regulation_of_protein_metabolic_process	795	962	1216	0.199198	0.0107	0.463	0.162
GO:0009966:regulation_of_signal_transduction	960	1394	1736	0.200048	0.0098	0.488	0.16
GO:0022838:substrate-specific_channel_activity	321	520	702	0.200089	0.0128	0.463	0.142
GO:0044093:positive_regulation_of_molecular_function	649	824	1033	0.200122	0.0126	0.485	0.159
GO:0065008:regulation_of_biological_quality	1392	2000	2390	0.200235	0.0076	0.511	0.181
GO:0019899:enzyme_binding	588	887	1023	0.200511	0.0121	0.534	0.161
GO:0010605:negative_regulation_of_macromolecule_metabolic_	668	704	919	0.200531	0.0146	0.414	0.165
GO:0071822:protein_complex_subunit_organization	418	529	670	0.200659	0.0169	0.467	0.175
GO:0009892:negative_regulation_of_metabolic_process	713	736	969	0.200827	0.0137	0.403	0.171
GO:0015267:channel_activity	332	533	710	0.201019	0.0128	0.474	0.141
GO:0022803:passive_transmembrane_transporter_activity	332	533	710	0.201019	0.0128	0.474	0.141
GO:0010941:regulation_of_cell_death	762	986	1184	0.201134	0.0115	0.514	0.162
GO:0070271:protein_complex_biogenesis	347	504	623	0.201332	0.0171	0.488	0.175
GO:0006928:cellular_component_movement	532	920	1050	0.201386	0.0124	0.56	0.141
GO:0006793:phosphorus_metabolic_process	577	758	997	0.201441	0.0129	0.412	0.183
GO:0006796:phosphate-containing_compound_metabolic_proces	577	758	997	0.201441	0.0129	0.412	0.183
GO:0008092:cytoskeletal_protein_binding	400	767	857	0.201502	0.0136	0.541	0.166
GO:0050878:regulation_of_body_fluid_levels	394	524	704	0.201524	0.0145	0.429	0.197
GO:0005829:cytosol	1418	1788	2228	0.201573	0.0079	0.465	0.174
GO:0007166:cell_surface_receptor_linked_signaling_pathway	1190	1659	2128	0.201971	0.0084	0.446	0.168
GO:0051173:positive_regulation_of_nitrogen_compound_metabo	671	733	938	0.201979	0.0139	0.444	0.158
GO:0007010:cytoskeleton_organization	374	658	757	0.202404	0.0146	0.537	0.17
GO:0043067:regulation_of_programmed_cell_death	739	953	1138	0.202407	0.0116	0.519	0.165
GO:0006461:protein_complex_assembly	345	503	621	0.202427	0.0172	0.488	0.176
GO:0051254:positive_regulation_of_RNA_metabolic_process	594	630	817	0.202828	0.0151	0.44	0.165
GO:0042981:regulation_of_apoptosis	733	946	1126	0.202993	0.0117	0.52	0.165
GO:0045935:positive_regulation_of_nucleobase-containing_comp	655	716	917	0.203097	0.0142	0.444	0.161
GO:0007599:hemostasis	334	462	619	0.204117	0.015	0.413	0.21
GO:0051172:negative_regulation_of_nitrogen_compound_metabo	487	518	660	0.204261	0.0153	0.437	0.174
GO:0007596:blood_coagulation	331	461	615	0.204366	0.015	0.415	0.21
GO:0050817:coagulation	331	461	615	0.204366	0.015	0.415	0.21
GO:0051049:regulation_of_transport	540	718	889	0.204599	0.0138	0.481	0.153
GO:0022892:substrate-specific_transporter_activity	742	1178	1446	0.204718	0.01	0.531	0.158
GO:0051094:positive_regulation_of_developmental_process	390	486	590	0.204832	0.0158	0.513	0.165
GO:0042060:wound_healing	408	599	747	0.206088	0.0136	0.465	0.202
GO:0065003:macromolecular_complex_assembly	464	632	771	0.206849	0.0153	0.476	0.167
GO:0032989:cellular_component_morphogenesis	490	809	985	0.206885	0.0118	0.493	0.177
GO:0032879:regulation_of_localization	742	1083	1305	0.207358	0.0119	0.488	0.178
GO:0045934:negative_regulation_of_nucleobase-containing_com	482	514	650	0.207878	0.0155	0.437	0.175
GO:0042592:homeostatic_process	646	925	1076	0.208832	0.0113	0.531	0.181
GO:0006357:regulation_of_transcription_from_RNA_polymerase_	652	658	837	0.208942	0.0149	0.452	0.169
GO:0046907:intracellular_transport	508	691	782	0.209389	0.0131	0.531	0.14
GO:0048878:chemical_homeostasis	450	625	759	0.209576	0.0147	0.501	0.196
GO:0051253:negative_regulation_of_RNA_metabolic_process	450	471	605	0.20991	0.0163	0.427	0.177
GO:2000113:negative_regulation_of_cellular_macromolecule_bio	483	506	642	0.209948	0.0174	0.426	0.183
GO:0048523:negative_regulation_of_cellular_process	1545	1956	2411	0.209978	0.0091	0.463	0.183

GO:0010629:negative_regulation_of_gene_expression	483	509	661	0.210373	0.0164	0.413	0.178
GO:0045893:positive_regulation_of_transcription,_DNA-depende	566	619	778	0.210427	0.016	0.449	0.17
GO:0022857:transmembrane_transporter_activity	700	1099	1343	0.21087	0.0105	0.521	0.166
GO:0055086:nucleobase-containing_small_molecule_metabolic_	397	495	572	0.2109	0.0185	0.519	0.168
GO:0031327:negative_regulation_of_cellular_biosynthetic_proces	518	540	685	0.210986	0.0167	0.423	0.183
GO:0043933:macromolecular_complex_subunit_organization	564	709	864	0.211059	0.0153	0.467	0.177
GO:0000267:cell_fraction	811	1328	1538	0.211105	0.0098	0.552	0.174
GO:0006811:ion_transport	591	979	1205	0.211152	0.0109	0.51	0.162
GO:0006996:organelle_organization	1078	1604	1810	0.211289	0.0095	0.538	0.172
GO:0005626:insoluble_fraction	610	1085	1246	0.211392	0.0108	0.565	0.169
GO:0045892:negative_regulation_of_transcription,_DNA-depende	440	458	592	0.212085	0.0166	0.415	0.182
GO:0009890:negative_regulation_of_biosynthetic_process	529	555	696	0.212097	0.0167	0.429	0.181
GO:0005654:nucleoplasm	855	971	1180	0.212153	0.0118	0.479	0.175
GO:0050790:regulation_of_catalytic_activity	866	1151	1383	0.212214	0.0118	0.493	0.17
GO:0031975:envelope	507	639	748	0.212384	0.0168	0.479	0.176
GO:0051641:cellular_localization	846	1266	1402	0.212522	0.0098	0.543	0.164
GO:0030234:enzyme_regulator_activity	648	1002	1153	0.212706	0.0115	0.534	0.161
GO:0016070:RNA_metabolic_process	822	935	1055	0.212885	0.0137	0.557	0.171
GO:0006464:protein_modification_process	1116	1478	1779	0.212917	0.009	0.47	0.182
GO:0044267:cellular_protein_metabolic_process	1558	1965	2291	0.213473	0.008	0.497	0.18
GO:0050793:regulation_of_developmental_process	787	1075	1278	0.213508	0.0109	0.503	0.202
GO:0065009:regulation_of_molecular_function	1063	1375	1631	0.213999	0.0102	0.503	0.175
GO:0048522:positive_regulation_of_cellular_process	1750	2222	2666	0.214074	0.0087	0.484	0.174
GO:0051276:chromosome_organization	380	577	600	0.214198	0.0172	0.628	0.174
GO:0048518:positive_regulation_of_biological_process	1927	2471	2939	0.214277	0.0083	0.49	0.183
GO:0010558:negative_regulation_of_macromolecule_biosynthetic	503	537	670	0.214311	0.0173	0.434	0.183
GO:0005624:membrane_fraction	587	1067	1225	0.21492	0.011	0.56	0.174
GO:2000026:regulation_of_multicellular_organismal_developmen	617	772	957	0.215249	0.0125	0.476	0.209
GO:0016043:cellular_component_organization	2173	3361	3861	0.215321	0.0068	0.519	0.186
GO:0071842:cellular_component_organization_at_cellular_level	1765	2680	3055	0.215434	0.0073	0.52	0.183
GO:0044431:Golgi_apparatus_part	409	491	596	0.215459	0.0169	0.456	0.179
GO:0009057:macromolecule_catabolic_process	377	494	540	0.215998	0.0187	0.533	0.208
GO:0051336:regulation_of_hydrolase_activity	416	536	675	0.216314	0.0157	0.453	0.161
GO:0030554:adenyl_nucleotide_binding	1172	2292	2444	0.216788	0.0085	0.602	0.172
GO:0032559:adenyl_ribonucleotide_binding	1165	2290	2439	0.21689	0.0085	0.603	0.171
GO:0016023:cytoplasmic_membrane-bounded_vesicle	498	786	898	0.216929	0.0151	0.551	0.192
GO:0007154:cell_communication	2634	3667	4325	0.217105	0.0062	0.501	0.179
GO:0019904:protein_domain_specific_binding	324	504	597	0.217243	0.0151	0.495	0.164
GO:0048519:negative_regulation_of_biological_process	1705	2196	2655	0.217574	0.0089	0.469	0.186
GO:0044463:cell_projection_part	391	799	836	0.217609	0.0134	0.637	0.185
GO:0048583:regulation_of_response_to_stimulus	1288	1858	2220	0.21818	0.0087	0.486	0.195
GO:0046483:heterocycle_metabolic_process	484	655	714	0.218251	0.0157	0.568	0.191
GO:0007165:signal_transduction	2245	3141	3689	0.218292	0.0067	0.5	0.177
GO:0043412:macromolecule_modification	1187	1611	1882	0.218371	0.0092	0.486	0.184
GO:0023052:signaling	2530	3537	4163	0.218855	0.0064	0.496	0.182
GO:0043234:protein_complex	2051	2948	3312	0.218863	0.0084	0.525	0.186
GO:0071310:cellular_response_to_organic_substance	543	687	815	0.218995	0.014	0.473	0.229
GO:0008283:cell_proliferation	321	495	551	0.219151	0.0182	0.597	0.148
GO:0032553:ribonucleotide_binding	1433	2560	2758	0.219178	0.0079	0.589	0.173

GO:0032555:purine_ribonucleotide_binding	1432	2559	2757	0.219185	0.0079	0.589	0.173
GO:0017076:purine_nucleotide_binding	1441	2566	2763	0.219372	0.0079	0.589	0.173
GO:0005524:ATP_binding	1145	2254	2399	0.219975	0.0087	0.598	0.175
GO:0005215:transporter_activity	912	1497	1739	0.220082	0.0094	0.554	0.173
GO:0051128:regulation_of_cellular_component_organization	662	967	1088	0.22018	0.0132	0.522	0.191
GO:0031410:cytoplasmic_vesicle	531	841	951	0.220713	0.0148	0.552	0.192
GO:0051649:establishment_of_localization_in_cell	718	1057	1142	0.220887	0.0119	0.554	0.161
GO:0010628:positive_regulation_of_gene_expression	604	677	856	0.22089	0.0155	0.432	0.173
GO:0035639:purine_ribonucleoside_triphosphate_binding	1409	2514	2712	0.220916	0.008	0.584	0.175
GO:0051726:regulation_of_cell_cycle	453	608	673	0.221173	0.0188	0.5	0.179
GO:0031967:organelle_envelope	496	611	695	0.221443	0.0179	0.484	0.185
GO:0071841:cellular_component_organization_or_biogenesis_at_	1826	2817	3153	0.22161	0.0071	0.526	0.186
GO:0010033:response_to_organic_substance	996	1332	1528	0.222207	0.0104	0.514	0.201
GO:0071840:cellular_component_organization_or_biogenesis	2249	3522	3972	0.222241	0.0067	0.525	0.19
GO:0048731:system_development	2046	3090	3614	0.222675	0.0067	0.498	0.205
GO:0016192:vesicle-mediated_transport	484	782	860	0.222896	0.0135	0.579	0.183
GO:0031988:membrane-bounded_vesicle	509	812	922	0.223037	0.0149	0.549	0.196
GO:0031982:vesicle	554	890	996	0.224303	0.0142	0.563	0.192
GO:0030154:cell_differentiation	1457	2117	2537	0.224457	0.0073	0.475	0.211
GO:0042623:ATPase_activity,_coupled	221	501	526	0.224688	0.0205	0.573	0.192
GO:0032991:macromolecular_complex	2450	3393	3738	0.224789	0.0078	0.537	0.19
GO:0051179:localization	2410	3777	4179	0.224922	0.0069	0.555	0.185
GO:0010467:gene_expression	906	1018	1114	0.225655	0.0129	0.575	0.173
GO:0051239:regulation_of_multicellular_organismal_process	1025	1334	1616	0.225808	0.0101	0.462	0.211
GO:0000166:nucleotide_binding	1822	3033	3227	0.226313	0.0083	0.582	0.18
GO:0070887:cellular_response_to_chemical_stimulus	782	1004	1134	0.227288	0.0121	0.535	0.214
GO:0055085:transmembrane_transport	527	879	1033	0.227346	0.0132	0.527	0.195
GO:0008104:protein_localization	715	1088	1174	0.227617	0.0128	0.535	0.185
GO:0016740:transferase_activity	1386	2043	2261	0.227651	0.009	0.541	0.194
GO:0003700:sequence-specific_DNA_binding_transcription_factc	665	584	739	0.228105	0.0173	0.452	0.169
GO:0001071:nucleic_acid_binding_transcription_factor_activity	667	585	741	0.228318	0.0172	0.452	0.168
GO:0051716:cellular_response_to_stimulus	2814	4085	4502	0.229533	0.0064	0.539	0.191
GO:0009611:response_to_wounding	623	922	1075	0.23078	0.0123	0.5	0.197
GO:0051234:establishment_of_localization	2011	3049	3399	0.23107	0.0076	0.539	0.192
GO:0046983:protein_dimerization_activity	476	621	665	0.231154	0.0186	0.582	0.185
GO:0005794:Golgi_apparatus	690	914	1054	0.231344	0.0119	0.471	0.215
GO:0019538:protein_metabolic_process	1950	2700	2978	0.231454	0.0077	0.531	0.201
GO:0006810:transport	1981	2985	3339	0.231589	0.0076	0.537	0.193
GO:0031981:nuclear_lumen	1488	1974	2157	0.232298	0.0092	0.547	0.198
GO:0042221:response_to_chemical_stimulus	1689	2394	2696	0.232877	0.0087	0.531	0.206
GO:0044428:nuclear_part	1682	2230	2439	0.232908	0.0088	0.538	0.196
GO:0003008:system_process	983	1520	1731	0.233115	0.0107	0.52	0.221
GO:0012505:endomembrane_system	1038	1410	1562	0.233675	0.0113	0.523	0.213
GO:0009059:macromolecule_biosynthetic_process	857	1131	1119	0.233921	0.0146	0.638	0.177
GO:0048584:positive_regulation_of_response_to_stimulus	568	760	893	0.234049	0.015	0.466	0.238
GO:0007275:multicellular_organismal_development	2438	3758	4242	0.23469	0.0067	0.509	0.214
GO:0042802:identical_protein_binding	480	598	681	0.23482	0.0185	0.529	0.191
GO:0048513:organ_development	1445	2145	2453	0.234858	0.009	0.501	0.216
GO:0051171:regulation_of_nitrogen_compound_metabolic_proce	2191	2625	3026	0.235373	0.0081	0.477	0.201

GO:0009653:anatomical_structure_morphogenesis	1120	1728	2039	0.235593	0.0097	0.468	0.209
GO:0048869:cellular_developmental_process	1503	2262	2624	0.235675	0.0075	0.488	0.221
GO:0048856:anatomical_structure_development	2263	3473	3946	0.235684	0.0067	0.498	0.219
GO:0005730:nucleolus	748	1047	1118	0.236454	0.0124	0.567	0.194
GO:0009790:embryo_development	530	714	808	0.236583	0.0173	0.49	0.189
GO:0044260:cellular_macromolecule_metabolic_process	2621	3657	3792	0.236643	0.0075	0.571	0.199
GO:0031323:regulation_of_cellular_metabolic_process	2703	3252	3741	0.23721	0.0074	0.475	0.203
GO:0022607:cellular_component_assembly	729	1138	1268	0.237565	0.0133	0.51	0.199
GO:0031090:organelle_membrane	1387	1767	1934	0.237624	0.0109	0.531	0.202
GO:0045184:establishment_of_protein_localization	566	811	874	0.23803	0.0154	0.516	0.193
GO:0033036:macromolecule_localization	859	1415	1439	0.238077	0.0115	0.592	0.195
GO:0034645:cellular_macromolecule_biosynthetic_process	840	1107	1087	0.238483	0.015	0.636	0.182
GO:0050794:regulation_of_cellular_process	4960	6866	7585	0.238704	0.0054	0.519	0.199
GO:0080090:regulation_of_primary_metabolic_process	2680	3295	3752	0.239051	0.0073	0.483	0.203
GO:0019219:regulation_of_nucleobase-containing_compound_m	2141	2584	2951	0.239358	0.0083	0.482	0.204
GO:0060255:regulation_of_macromolecule_metabolic_process	2562	3114	3560	0.23973	0.0078	0.481	0.204
GO:0065007:biological_regulation	5554	7945	8623	0.239733	0.0052	0.532	0.203
GO:0007155:cell_adhesion	464	1100	1213	0.240373	0.0123	0.545	0.222
GO:0022610:biological_adhesion	464	1100	1213	0.240373	0.0123	0.545	0.222
GO:0009628:response_to_abiotic_stimulus	370	609	625	0.240543	0.0174	0.624	0.21
GO:0005515:protein_binding	4128	6043	6474	0.240993	0.0056	0.544	0.206
GO:0015031:protein_transport	557	793	847	0.241062	0.0156	0.523	0.197
GO:0044429:mitochondrial_part	453	546	542	0.241774	0.0195	0.615	0.177
GO:0005634:nucleus	3650	4751	5028	0.241798	0.0066	0.552	0.198
GO:0005102:receptor_binding	739	979	1022	0.241871	0.014	0.572	0.218
GO:0070013:intracellular_organelle_lumen	1734	2356	2498	0.242056	0.0096	0.564	0.202
GO:0050789:regulation_of_biological_process	5255	7414	8072	0.242179	0.0054	0.525	0.203
GO:0050877:neurological_system_process	755	1149	1296	0.242368	0.012	0.515	0.239
GO:0032501:multicellular_organismal_process	3362	5273	5854	0.242856	0.0061	0.517	0.221
GO:0009889:regulation_of_biosynthetic_process	2125	2567	2914	0.243318	0.0089	0.479	0.212
GO:0006508:proteolysis	588	920	951	0.243395	0.0148	0.589	0.206
GO:0032502:developmental_process	2662	4097	4565	0.243491	0.0066	0.505	0.226
GO:0031974:membrane-enclosed_lumen	1784	2449	2584	0.243969	0.0096	0.564	0.206
GO:0006139:nucleobase-containing_compound_metabolic_proce	1616	2225	2224	0.2442	0.0104	0.607	0.198
GO:0044459:plasma_membrane_part	1296	2358	2625	0.244269	0.0086	0.53	0.219
GO:0031326:regulation_of_cellular_biosynthetic_process	2109	2539	2884	0.244398	0.0091	0.476	0.214
GO:0043233:organelle_lumen	1757	2427	2561	0.244423	0.0096	0.565	0.206
GO:0019222:regulation_of_metabolic_process	2965	3718	4160	0.244557	0.0071	0.491	0.207
GO:0044444:cytoplasmic_part	4151	5736	6037	0.245252	0.0063	0.56	0.205
GO:0010556:regulation_of_macromolecule_biosynthetic_process	1991	2390	2703	0.246205	0.0093	0.476	0.217
GO:0044085:cellular_component_biogenesis	816	1285	1391	0.24838	0.0124	0.518	0.204
GO:0034641:cellular_nitrogen_compound_metabolic_process	1931	2677	2678	0.248763	0.0097	0.601	0.209
GO:0043170:macromolecule_metabolic_process	3074	4495	4572	0.248833	0.0072	0.582	0.211
GO:2000112:regulation_of_cellular_macromolecule_biosynthetic_	1951	2336	2631	0.249026	0.0096	0.476	0.22
GO:0044237:cellular_metabolic_process	3947	5532	5717	0.249179	0.0065	0.564	0.214
GO:0009987:cellular_process	7681	11623	12113	0.249603	0.0044	0.566	0.211
GO:0009058:biosynthetic_process	1455	1959	1977	0.250098	0.0113	0.59	0.213
GO:0051252:regulation_of_RNA_metabolic_process	1872	2211	2491	0.250145	0.0093	0.478	0.222
GO:2001141:regulation_of_RNA_biosynthetic_process	1835	2184	2461	0.250468	0.0096	0.476	0.223

GO:0044446:intracellular_organelle_part	3893	5466	5689	0.250566	0.0067	0.56	0.208
GO:0016887:ATPase_activity	281	776	712	0.250663	0.0175	0.698	0.2
GO:0010468:regulation_of_gene_expression	2059	2467	2784	0.250936	0.0093	0.476	0.218
GO:0044249:cellular_biosynthetic_process	1385	1828	1854	0.251064	0.0117	0.58	0.217
GO:0016817:hydrolase_activity,_acting_on_acid_anhydrides	619	1325	1282	0.251066	0.0135	0.631	0.207
GO:0016462:pyrophosphatase_activity	613	1325	1279	0.251238	0.0135	0.634	0.207
GO:0016818:hydrolase_activity,_acting_on_acid_anhydrides,_in_	616	1325	1279	0.251238	0.0135	0.634	0.207
GO:0005783:endoplasmic_reticulum	716	1036	1004	0.251474	0.0134	0.662	0.189
GO:0044248:cellular_catabolic_process	785	1108	1160	0.25198	0.0141	0.529	0.236
GO:0006355:regulation_of_transcription,_DNA-dependent	1824	2183	2450	0.252119	0.0096	0.477	0.225
GO:0043228:non-membrane-bounded_organelle	2236	3592	3657	0.253155	0.008	0.572	0.216
GO:0043232:intracellular_non-membrane-bounded_organelle	2236	3592	3657	0.253155	0.008	0.572	0.216
GO:0016874:ligase_activity	334	511	538	0.253245	0.0205	0.54	0.228
GO:0009887:organ_morphogenesis	484	687	773	0.2533	0.0167	0.485	0.211
GO:0044422:organelle_part	3948	5626	5828	0.253338	0.0068	0.563	0.212
GO:0005856:cytoskeleton	1142	2143	2195	0.253416	0.0112	0.558	0.221
GO:0006807:nitrogen_compound_metabolic_process	2017	2829	2806	0.253549	0.0096	0.607	0.217
GO:0005737:cytoplasm	5695	8116	8373	0.254054	0.0055	0.565	0.21
GO:0017111:nucleoside-triphosphatase_activity	589	1295	1246	0.254382	0.0137	0.627	0.213
GO:0043231:intracellular_membrane-bounded_organelle	5940	8092	8225	0.255142	0.0056	0.575	0.212
GO:0043227:membrane-bounded_organelle	5945	8099	8234	0.255291	0.0056	0.575	0.212
GO:0044238:primary_metabolic_process	4098	6023	6096	0.255464	0.0068	0.579	0.221
GO:0048646:anatomical_structure_formation_involved_in_morph	393	626	695	0.257011	0.0189	0.506	0.228
GO:0071844:cellular_component_assembly_at_cellular_level	528	874	893	0.257216	0.0159	0.554	0.219
GO:0044424:intracellular_part	7869	11080	11205	0.257478	0.0049	0.579	0.213
GO:0005622:intracellular	8231	11661	11781	0.257763	0.0049	0.581	0.212
GO:0043229:intracellular_organelle	6692	9419	9502	0.257892	0.0055	0.577	0.215
GO:0043226:organelle	6700	9432	9516	0.257978	0.0055	0.577	0.215
GO:0002376:immune_system_process	885	1169	1261	0.258273	0.0138	0.494	0.249
GO:0008152:metabolic_process	4604	6735	6780	0.259408	0.0063	0.579	0.224
GO:0005975:carbohydrate_metabolic_process	451	737	724	0.260228	0.0188	0.581	0.213
GO:0005887:integral_to_plasma_membrane	762	1341	1519	0.260415	0.0122	0.49	0.234
GO:0031226:intrinsic_to_plasma_membrane	788	1374	1549	0.260551	0.012	0.496	0.233
GO:0090304:nucleic_acid_metabolic_process	1234	1755	1647	0.260817	0.0128	0.645	0.216
GO:0044281:small_molecule_metabolic_process	1272	1944	1922	0.261145	0.0108	0.619	0.229
GO:0009888:tissue_development	672	1054	1174	0.261508	0.0127	0.474	0.261
GO:0009056:catabolic_process	962	1393	1404	0.261989	0.0134	0.558	0.251
GO:0003723:RNA_binding	546	577	623	0.262546	0.0244	0.506	0.216
GO:0046872:metal_ion_binding	2833	4823	4742	0.26318	0.0065	0.61	0.223
GO:0022414:reproductive_process	741	1144	1098	0.26427	0.0141	0.623	0.217
GO:0000278:mitotic_cell_cycle	363	522	592	0.264822	0.0201	0.414	0.265
GO:0071702:organic_substance_transport	272	567	544	0.264906	0.0174	0.624	0.284
GO:0000003:reproduction	744	1146	1104	0.265257	0.0141	0.616	0.218
GO:0005488:binding	8391	12913	12759	0.266124	0.0046	0.594	0.225
GO:0044432:endoplasmic_reticulum_part	481	681	657	0.26626	0.0178	0.639	0.225
GO:0043167:ion_binding	2876	4916	4809	0.266348	0.0065	0.611	0.225
GO:0043169:cation_binding	2870	4910	4797	0.267127	0.0066	0.611	0.225
GO:0042175:nuclear_outer_membrane-endoplasmic_reticulum_n	445	614	598	0.267346	0.0196	0.621	0.24
GO:0005789:endoplasmic_reticulum_membrane	431	597	579	0.267848	0.0199	0.631	0.235

GO:0003824:catalytic_activity	4151	6849	6751	0.268096	0.006	0.592	0.238
GO:0005739:mitochondrion	980	1169	1117	0.268658	0.0155	0.613	0.21
GO:0044430:cytoskeletal_part	835	1596	1605	0.269157	0.0133	0.562	0.236
GO:0006066:alcohol_metabolic_process	312	526	502	0.269607	0.0208	0.606	0.248
GO:0003676:nucleic_acid_binding	2365	3028	3001	0.269625	0.0097	0.575	0.224
GO:0080134:regulation_of_response_to_stress	390	538	582	0.271493	0.0197	0.479	0.334
GO:0003677:DNA_binding	1595	2062	2025	0.272903	0.0116	0.571	0.244
GO:0007049:cell_cycle	670	1052	1066	0.273023	0.015	0.521	0.23
GO:0009986:cell_surface	282	483	519	0.274604	0.0188	0.516	0.235
GO:0006950:response_to_stress	1611	2375	2401	0.27478	0.0108	0.547	0.266
GO:0002682:regulation_of_immune_system_process	489	611	686	0.276352	0.0179	0.421	0.342
GO:0007017:microtubule-based_process	217	534	492	0.277274	0.0217	0.617	0.236
GO:0008270:zinc_ion_binding	1489	2345	2202	0.27741	0.0101	0.63	0.225
GO:0004175:endopeptidase_activity	303	522	527	0.278657	0.0193	0.582	0.262
GO:0070011:peptidase_activity,_acting_on_L-amino_acid_peptid	433	763	739	0.279801	0.0163	0.632	0.249
GO:0055114:oxidation-reduction_process	402	608	580	0.280096	0.0224	0.604	0.247
GO:0008233:peptidase_activity	457	800	762	0.280242	0.016	0.653	0.24
GO:0006974:response_to_DNA_damage_stimulus	332	538	482	0.280322	0.0236	0.636	0.301
GO:0016491:oxidoreductase_activity	554	826	741	0.280546	0.0168	0.66	0.246
GO:0046914:transition_metal_ion_binding	1708	2755	2551	0.281469	0.0092	0.647	0.232
GO:0048609:multicellular_organismal_reproductive_process	391	662	604	0.28321	0.0202	0.637	0.26
GO:0032504:multicellular_organism_reproduction	391	662	604	0.28321	0.0202	0.637	0.26
GO:0022402:cell_cycle_process	535	895	873	0.285577	0.0162	0.529	0.256
GO:0005623:cell	10748	16357	15816	0.28687	0.0045	0.588	0.242
GO:0044464:cell_part	10747	16356	15813	0.286958	0.0045	0.588	0.242
GO:0033554:cellular_response_to_stress	575	867	814	0.287918	0.0177	0.589	0.298
GO:0009308:amine_metabolic_process	390	576	577	0.289057	0.0233	0.55	0.332
GO:0008150:biological_process	10243	16043	15459	0.289405	0.0046	0.587	0.248
GO:0016787:hydrolase_activity	1810	3440	3201	0.290579	0.0089	0.621	0.263
GO:0003674:molecular_function	11210	17637	16817	0.292659	0.0044	0.594	0.252
GO:0005575:cellular_component	11522	17552	16688	0.293881	0.0046	0.596	0.252
GO:0016020:membrane	5195	8539	8324	0.293983	0.0063	0.574	0.258
GO:0022403:cell_cycle_phase	438	738	730	0.296054	0.0185	0.504	0.278
GO:0005509:calcium_ion_binding	492	1107	1021	0.296544	0.0155	0.612	0.254
GO:0015630:microtubule_cytoskeleton	528	1074	1005	0.296609	0.0171	0.57	0.25
GO:0019953:sexual_reproduction	352	675	598	0.298196	0.0198	0.682	0.25
GO:0050896:response_to_stimulus	4220	6821	6620	0.29923	0.007	0.563	0.263
GO:0007276:gamete_generation	304	562	497	0.299358	0.0224	0.651	0.279
GO:0006629:lipid_metabolic_process	657	1064	985	0.301865	0.0168	0.62	0.292
GO:0051704:multi-organism_process	592	824	838	0.304038	0.0198	0.496	0.294
GO:0005615:extracellular_space	526	892	801	0.306037	0.0159	0.651	0.302
GO:0031012:extracellular_matrix	297	744	754	0.307854	0.018	0.532	0.319
GO:0071944:cell_periphery	2714	5061	4943	0.308239	0.0084	0.563	0.283
GO:0005886:plasma_membrane	2666	5006	4891	0.309227	0.0085	0.56	0.287
GO:0005694:chromosome	364	572	548	0.311907	0.0235	0.565	0.259
GO:0043436:oxoacid_metabolic_process	515	814	746	0.313308	0.0201	0.631	0.338
GO:0019752:carboxylic_acid_metabolic_process	515	814	746	0.313308	0.0201	0.631	0.338
GO:0044425:membrane_part	4128	7060	6614	0.313475	0.007	0.588	0.277
GO:0006082:organic_acid_metabolic_process	526	845	766	0.313711	0.0195	0.649	0.333

GO:0006259:DNA_metabolic_process	485	911	715	0.314683	0.0217	0.751	0.264
GO:0042180:cellular_ketone_metabolic_process	528	833	760	0.316727	0.0206	0.627	0.339
GO:0044421:extracellular_region_part	725	1395	1298	0.319267	0.0133	0.593	0.342
GO:0016788:hydrolase_activity,_acting_on_ester_bonds	527	948	847	0.319903	0.0182	0.586	0.376
GO:0005578:proteinaceous_extracellular_matrix	257	684	678	0.320989	0.0203	0.539	0.329
GO:0005198:structural_molecule_activity	464	848	769	0.323	0.0205	0.592	0.307
GO:0000279:M_phase	283	544	488	0.323493	0.0234	0.576	0.284
GO:0030246:carbohydrate_binding	272	534	495	0.327963	0.0256	0.553	0.289
GO:0031224:intrinsic_to_membrane	3569	6168	5655	0.332252	0.008	0.585	0.295
GO:0016021:integral_to_membrane	3497	6088	5558	0.334064	0.008	0.586	0.297
GO:0007600:sensory_perception	313	604	500	0.335213	0.0243	0.719	0.312
GO:0044255:cellular_lipid_metabolic_process	475	714	679	0.339635	0.022	0.519	0.347
GO:0005815:microtubule_organizing_center	277	542	484	0.34376	0.0249	0.5	0.399
GO:0005576:extracellular_region	1322	2281	1948	0.345448	0.0122	0.644	0.341
GO:0006952:defense_response	525	764	691	0.350595	0.0224	0.556	0.345
GO:0006955:immune_response	500	696	625	0.356863	0.0232	0.533	0.393
GO:0004871:signal_transducer_activity	1170	2350	1994	0.400144	0.0161	0.586	0.369
GO:0004872:receptor_activity	1233	2710	2218	0.405647	0.0148	0.615	0.368
GO:0038023:signaling_receptor_activity	913	2046	1583	0.455977	0.0191	0.624	0.43
GO:0004888:transmembrane_signaling_receptor_activity	844	1991	1489	0.465668	0.0198	0.654	0.437
GO:0004930:G-protein_coupled_receptor_activity	604	1469	890	0.60268	0.0293	0.741	0.573
GO:0004984:olfactory_receptor_activity	310	1024	430	0.895853	0.0478	1.011	0.993
